# Tuning Aqueous Supramolecular Polymerization by an Acid‐Responsive Conformational Switch

**DOI:** 10.1002/chem.202001566

**Published:** 2020-07-14

**Authors:** Christina Rest, Divya Susan Philips, Torsten Dünnebacke, Papri Sutar, Angel Sampedro, Jörn Droste, Vladimir Stepanenko, Michael Ryan Hansen, Rodrigo Q. Albuquerque, Gustavo Fernández

**Affiliations:** ^1^ Institut für Organische Chemie Universität Würzburg am Hubland 97078 Würzburg Germany; ^2^ Organisch-Chemisches Institut Westfälische Wilhelms-Universität (WWU) Münster Corrensstraße, 40. 48149 Münster Germany; ^3^ Institut für Physikalische Chemie WWU Münster Corrensstraße, 28/30 48149 Münster Germany

**Keywords:** acid-sensitive, amphiphilic systems, π-conjugated systems, noncovalent interactions, self-assembly

## Abstract

Besides their widespread use in coordination chemistry, 2,2’‐bipyridines are known for their ability to undergo *cis–trans* conformational changes in response to metal ions and acids, which has been primarily investigated at the molecular level. However, the exploitation of such conformational switching in self‐assembly has remained unexplored. In this work, the use of 2,2’‐bipyridines as acid‐responsive conformational switches to tune supramolecular polymerization processes has been demonstrated. To achieve this goal, we have designed a bipyridine‐based linear bolaamphiphile, **1**, that forms ordered supramolecular polymers in aqueous media through cooperative aromatic and hydrophobic interactions. Interestingly, addition of acid (TFA) induces the monoprotonation of the 2,2’‐bipyridine moiety, leading to a switch in the molecular conformation from a linear (*trans*) to a V‐shaped (*cis*) state. This increase in molecular distortion along with electrostatic repulsions of the positively charged bipyridine‐H^+^ units attenuate the aggregation tendency and induce a transformation from long fibers to shorter thinner fibers. Our findings may contribute to opening up new directions in molecular switches and stimuli‐responsive supramolecular materials.

## Graphical abstract

**Switch and tune**! The *trans*–*cis* conformational switching of 2,2’‐bipyridine in response to acid was exploited to tune supramolecular polymerization processes. For this purpose, the aqueous self‐assembly of a bipyridine‐based linear bolaamphiphile that self‐assembles under standard conditions into cooperative supramolecular polymers was investigated. Interestingly, acid‐triggered protonation of the bipyridine unit induces a molecular conformational change from linear (*trans*) to *cis* (V‐shaped) that leads to an attenuated supramolecular growth into shorter fibers.

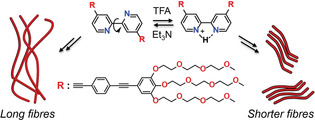

## Introduction

Subtle conformational changes of biomacromolecules, triggered by physiological stimuli, regulate various complex biological events, such as protein folding and membrane transport.^1^ Chemists have long sought to replicate these phenomena by synthesizing artificial molecular switches^2^ and machines^3^ that show switching between two conformational states in response to external stimuli, such as light,2b redox,2c pH,2d or cation/anions.2e In recent years, different types of molecular switches derived from diarylethenes,4a spiropyrans,4b spirooxazines,4c fulgides,4d and flavylium4e have been widely explored, with some promising applications in bioimaging,5a drug delivery,5b organic light‐emitting diodes,5c molecular electronics5d and catalysis.5e In this regard, 2,2’‐bipyridine represents an archetypal example of a molecular switch that can change its conformation from the linear *trans*‐state to the V‐shaped *cis*‐state by acid‐induced protonation as well as by metal complexation.^6^ To date, conformational switching of 2,2’‐bipyridines has been mainly investigated at the molecular level, for example to obtain molecular hinges7a and cavitands.7b However, to the best of our knowledge, bipyridines have not been exploited as conformational switches in supramolecular polymerization. We foresee that this concept would not only broaden the range of applications of bipyridines but also complement the existing arsenal of tools in stimuli‐responsive supramolecular materials.

In this regard, exploiting molecular conformational changes in response to external stimuli is a promising way to tune self‐assembly processes and create smart materials.^8^ The most commonly used method to achieve conformational switching is the use of light as external trigger, which allows precise control over the dimensionality and properties of supramolecular assemblies.^9^ In contrast, the effect of ion‐induced conformational changes on supramolecular polymerization has been comparatively less studied, for which 2,2’‐bipyridine would be an ideal candidate. To date, research on bipyridine‐based supramolecular polymers has been limited to coordination polymers,10a host–guest stimuli‐responsive supramolecular polymers,10b, 10c, 10d and cross‐linked supramolecular polymers.10e, 10f However, understanding how the ion‐induced changes in molecular shape (*trans* vs. *cis*) affects self‐assembly of bipyridine‐based systems remains elusive.

**Scheme 1:**
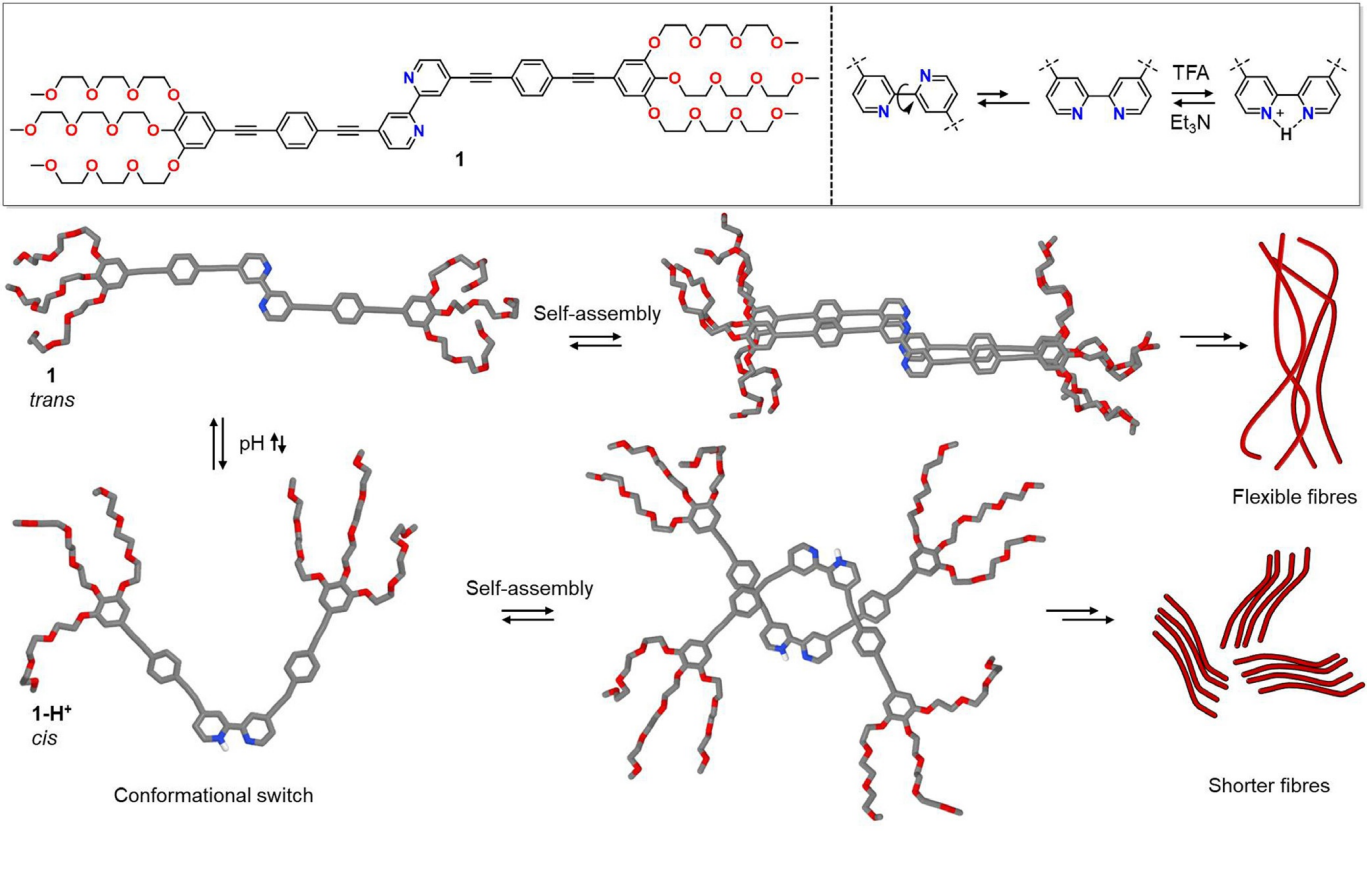
Top: Molecular structure of **1** and schematic representation of its conformational switching in response to addition of acid and base. Bottom: Self‐assembly of *trans*‐**1** and *cis*‐locked **1‐H^+^** into long bundles of flexible fibers and shorter thinner fibers, respectively. The monomer and dimer of *trans*‐**1** and *cis*‐locked **1‐H^+^** are dispersion‐corrected PM6‐optimized structures.

Herein, we demonstrate the use of 2,2’‐bipyridines as acid‐responsive conformational switches to tune aqueous self‐assembly processes.^11^ To this end, we have designed a bolaamphiphilic derivative **1**, in which a central 2,2’‐bipyridine unit is conjugated at the 4,4’‐positions with oligophenyleneethynylene (OPE) fragments bearing hydrophilic triethyleneglycol (TEG) chains (Scheme 1, for synthesis and characterization, see the Supporting Information) Interestingly, we found that the acid‐induced *trans*‐to‐*cis* conformational change of the 2,2’‐bipyridine unit, along with the electrostatic repulsion resulting from protonation, immensely impact the molecular packing and attenuate the aggregation tendency of **1**, leading to a transformation from long fibers to shorter thinner fibers.

## Results and Discussion

### Aqueous supramolecular polymerization of 1

**Figure 1:**
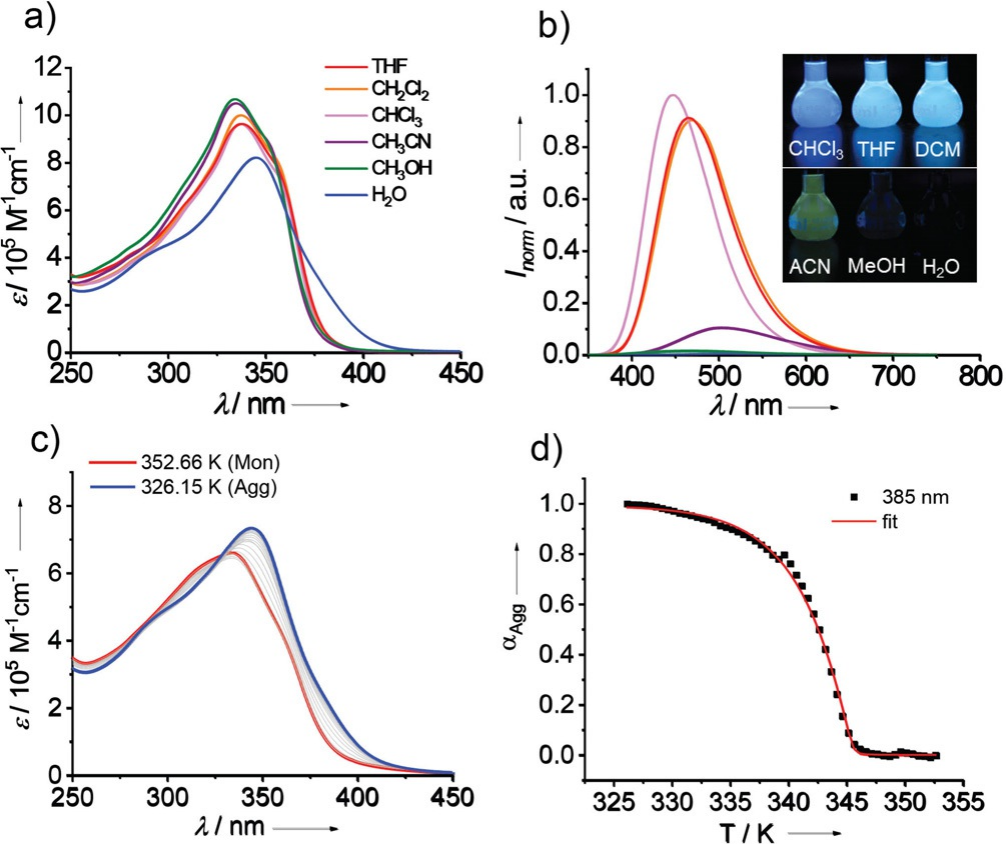
Solvent‐dependent UV/Vis absorption (a) and emission studies (b) of compound **1** (inset: photograph illustrating the luminescence of compound **1** in different solvents). *λ*_exc_: 335 nm. (c) Temperature‐dependent UV/Vis absorption spectra of **1** in water (2.0×10^−5^ m) upon cooling from 353 K to 326 K using a rate of 0.1 K min^−1^. (d) Cooling curve obtained by monitoring absorption changes at 385 nm and their fitting to the cooperative self‐assembly model (red line).

Ligand **1** is readily soluble in moderately polar solvents, such as THF, dichloromethane and chloroform, suggesting a molecularly dissolved state in these media. However, in more polar, protic solvents such as methanol and water, the solubility is decreased, which is a first sign of aggregation. Figure 1 a shows the solvent‐dependent UV/Vis absorption measurements of **1** at *c=*1×10^−5^ m. As expected, the absorption spectra in most organic solvents exhibit a similar shape with a maximum centered at ≈336 nm, characteristic of a monomeric state. In aqueous solution, however, the maximum is redshifted to 345 nm and the spectrum broadens up to 425 nm suggesting strong intermolecular interactions of the OPE core. The solvent‐dependent emission characteristics of **1** were investigated under the same conditions (Figure 1 b and Figure S1). The maximum in chloroform is located at 448 nm, while it shifts to ≈467 nm in THF and dichloromethane. In contrast, the emission intensity is significantly reduced in acetonitrile with a maximum at 503 nm, while it is almost quenched in methanol and water. This indicates a clear solvatochromism for **1** that is typical in donor–acceptor–donor systems.6e With increasing polarity, there is a bathochromic shift in the emission maximum (Figure S1). The observed difference in intensity in emission in polar solvents like acetonitrile, methanol and water can be understood from the differences in solubility of the molecule in these media as well as by the use of the same excitation wavelength (335 nm) for all solvents. The relative emission of **1** in different solvents becomes evident when comparing photographs of the respective solutions under UV light (inset of Figure 1 b). The quantum yields were determined in different solvents using quinine sulphate as the standard,^12^ and the values are shown in Table S1.

To unravel the aggregation mechanism of **1** in solution, variable‐temperature (VT) absorption measurements were performed. Since the spectral features in different solvents clearly suggest a high aggregation tendency of amphiphile **1** in aqueous environment, this medium was chosen to investigate the self‐assembly. Besides pure water, a solvent mixture containing 1 % of THF was used to facilitate the sample preparation. This preparation protocol allows to create stable aggregate solutions with invariant absorption spectra over the course of several weeks, so that possible kinetic effects can be discarded. The temperature‐dependent studies of **1** at different concentrations (1.5–2.9×10^−5^ m) in both water and water/THF 99:1 resulted in identical spectral changes (Figure S2), indicating that the small amount of THF has no significant influence on the self‐assembly process. To understand the aggregation mechanism, a thin film of compound **1** (prepared by evaporation of 30 μL stock solution in THF) was dissolved in water (*c=*2.0×10^−5^ m) and investigated by VT UV/Vis measurements (Figure 1 c). The sample was heated to its molecularly dissolved state (353 K) and afterwards cooled down slowly (0.1 K min^−1^) to ensure aggregation under thermodynamic control. It is worth noting that we did not observe a lower critical solution temperature (LCST) upon heating the sample. Upon cooling to 326 K, the absorption maximum undergoes a gradual redshift from 334 to 346 nm through an isosbestic point at 325 nm. Additionally, the overall absorption slightly increases and a new shoulder band at ≈385 nm is observed upon cooling (Figure 1 c). To analyze the underlying aggregation mechanism, the spectral changes at 385 nm were monitored as a function of temperature. The resulting cooling curves (plots of fraction of aggregated species (*α*_agg_) against temperature) reveal a non‐sigmoidal plot, characteristic of a cooperative supramolecular polymerization (Figure 1 d). Particularly relevant is the fact that the cooling curves do not appear to reach a clear plateau when the aggregation is presumably complete (Figure S3). A similar observation was reported by Meijer and co‐workers for the self‐assembly of hydrogen‐bonded OPVs, and was attributed to a gradual conversion of the already formed one‐dimensional structures into bundled assemblies.^13^ To facilitate the curve analysis, the non‐sigmoidal plots were fitted to the cooperative nucleation‐elongation model.^14^ The thermodynamic parameters were determined as follows: elongation temperature (*T_e_*)=345 K; elongation enthalpy (Δ*H*_e_)=−262.35 kJ mol^−1^; Gibbs free energy (Δ*G*_298_)=−62.69 kJ mol^−1^ and the degree of cooperativity (*σ*)=8.4×10^−4^ (Table S2). The cooperative nature of the self‐assembly of **1** was also supported by UV/Vis denaturation experiments,^15^ where the supramolecular polymers of **1** in water were gradually disassembled by addition of increasing aliquots of monomeric **1** in THF (Figure S4 and Table S3). Additionally, the initial stages of the aggregation process (nucleation) of **1** were also monitored by diffusion ordered spectroscopy (DOSY) NMR experiments at variable temperatures (Figures S5 and S6).

### Molecular conformation and morphology of 1 by solid‐state NMR analysis

**Figure 2:**
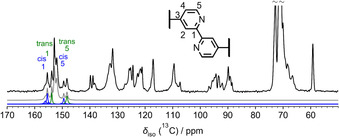
Solid‐state ^13^C{^1^H} CP/MAS NMR spectrum of **1** recorded at 9.4 T with a MAS frequency of 11.0 kHz. The inset shows the assignment of the bipyridine carbon atoms. The grey spectrum shows the complete deconvolution. The signals corresponding to *cis* and *trans* isomers are shown in blue and green, respectively. A full signal assignment of all ^13^C signals is given in Figure S8.

It has been widely established that bipyridine derivatives preferentially exist in the energetically most stable *transoid* conformation in solution in neutral media.^6^ In order to determine whether this is also the case for our system in its solid form, we performed solid‐state NMR experiments (Figure 2). The dried sample obtained after purification was used as such without any further treatment for solid state analysis to ensure that the system is in thermodynamic equilibrium. Specifically, we have taken advantage of the ^13^C isotropic chemical shift as a reporter of conformational differences by ^13^C{^1^H} CP/MAS NMR spectra as shown in Figure 2.16a The application of 2D ^1^H–^1^H double‐quantum single‐quantum (DQ‐SQ) and ^13^C{^1^H} heteronuclear correlation (HETCOR) experiments are then used to probe the aggregation and stacking behavior of **1**.16b From the overall line shape, width, and characteristic splitting of the ^13^C signals in Figure 2, it is evident that **1** contains two different species in the solid‐state, probably because we have used the dried sample as such without any further treatment. In particular, the ^13^C resolution in the region 145–160 ppm allows us to differentiate between the *cis* and *trans* isomers of **1**. The ^13^C signals that are most affected by a conformational change from *trans* to *cis*‐bipyridine are carbon signals 1 and 5, as highlighted in Figure 2. Both ^13^C signals are shifted to higher ppm values for the *cis* isomer as supported by gas‐phase DFT calculations of ^13^C chemical shifts for the *cis* and *trans* monomer (see Table S4). Furthermore, the calculations for **1**, where the glycol chains were substituted by methoxy groups (Figure S7), show that the *trans*‐form is energetically favored by 24.7 kJ mol^−1^, which is in excellent agreement with the energetic difference of non‐substituted 2,2’‐bipyridine.^6^ Despite this higher stability of the *trans* form, deconvolution of the ^13^C signals for carbon 5 reveals that the sample of **1** exists as a mixture of both *cis*‐ and *trans*‐conformations, however with a substantial preference for the *trans*‐state (*cis*/*trans*≈37:63). The formation of more than one type of packing for **1** in the solid‐state can also be identified in the 2D solid‐state NMR experiments (Figures S9 and S10). From the 2D ^13^C{^1^H} HETCOR NMR spectrum, the ^13^C signals from carbon positions 1 and 5 of *cis*‐**1** and *trans*‐**1** give rise to a number of different ^1^H positions (Figure S9). These can be grouped into sets of ^1^H signals for *trans*‐**1** that are located at lower ppm values compared to *cis*‐**1**. It can also be observed that the *trans*‐form undergoes more pronounced π–π stacking interactions, leading to a shift of the ^1^H due to aromatic ring current effects of neighboring molecules of **1**.16c, 16d Moreover, an autocorrelation ^1^H–^1^H signal of the proton bound to carbon 5 (cf. inset in Figure 2) can be observed in the 2D ^1^H–^1^H DQ‐SQ NMR correlation spectrum only for *trans*‐**1**, while this signal is missing for the *cis*‐**1** species (see red circle in Figure S10). These observations suggest the formation of 1D helical stacks for *trans*‐**1** with a rotational displacement of the monomer units along the fiber‐growing direction (see cartoon in Figure S14).16c On the other hand, the lack of this correlation signal for *cis*‐**1** suggests a different, possibly less ordered stacking pattern. Thus, the results from solid‐state NMR show that *cis*‐**1** and *trans*‐**1** are characterized by distinct aggregation behavior and molecular packing motifs, with *cis*‐**1** exhibiting less efficient aromatic interactions because of its more distorted conformation.

### Aggregate morphology of 1

**Figure 3:**
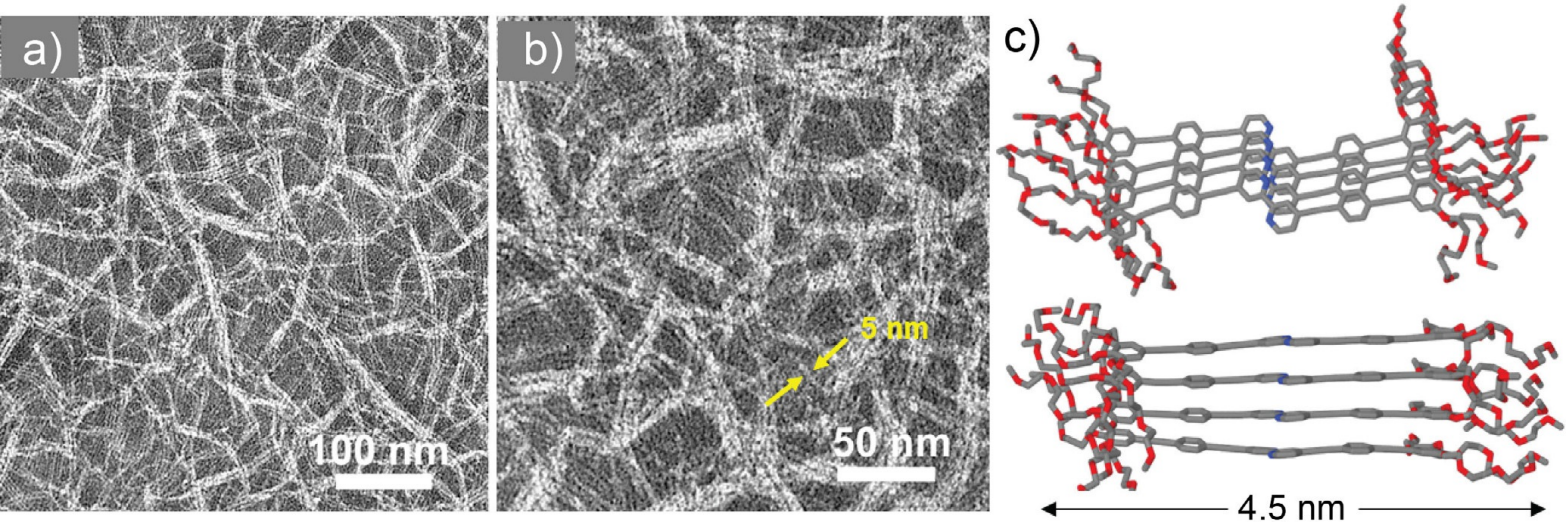
a, b) TEM image of **1** in aqueous solution after an aging time of few days (8.0×10^−4^ m) showing extended fiber formation. c) Scheme showing the optimized geometry of the tetramer with average length ≈4.5 nm, closely matching with the thickness of a single fiber.

The aggregate morphology of **1** was examined by transmission electron microscopy (TEM) on a carbon‐coated copper grid. The TEM studies of concentrated samples (5.7 and 8.0×10^−4^ m) of **1** in aqueous solution (Figure 3 a, b, and Figures S11 and S12) reveal flexible, well‐defined nanofibers with a highly uniform width of 4–5 nm. As evident from the images, the fibers are densely packed and have a strong tendency to bundle, as previously suggested by UV/Vis studies. It also becomes apparent that increasing the concentration of the aggregate solution leads to longer fibers, with a length that can be estimated to be several hundreds of nanometers. Given that the *trans*‐form of **1** is the major species formed in solution,^6^ we can assume that only this more stable and preorganized conformation is able to engage in supramolecular polymerization, whereas the less stable, distorted *cis*‐form is present, if at all, only in trace amounts. A similar behavior has been recently observed for self‐aggregating Pt^II^ complexes undergoing *cis‐trans* coordination isomerism,^17^ where the dormant *cis*‐isomer is completely removed from the equilibrium due to the stabilization of the aggregation‐active *trans*‐form by aggregation and solvation effects. A similar stabilization of the *trans*‐state is expected here.

### Quantum chemical calculations of 1

Based on the hypothesis that amphiphile **1** is primarily present in its linear *trans* conformation, we next aimed at rationalizing how the monomer units would pack into the 1D stacks. For this purpose, quantum chemical calculations at the dispersion‐corrected PM6 semiempirical level were performed. The optimized geometry of a monomer is shown in Scheme 1. The molecular length calculated for **1** (4.5 nm) is in excellent agreement with the experimental diameter of fibers of **1** (4–5 nm) measured by TEM, indicating that the supramolecular fibers are one molecule thick (Figure 3 c). Interdigitation of glycol chains would only be possible when two single fibers interact in a parallel fashion, giving rise to thicker bundles. The optimized geometry of the dimer and tetramer reveals that aromatic interactions take place along the whole π‐conjugated system of **1** and that a slight shift is observed along both the directions (X,Y) perpendicular to the long (Z) axis of the stack (see top view of dimer or tetramer in Scheme 1 and Figure S13). This slightly shifted packing is in accordance with a redshift in absorption upon aggregation.18a The interdisc distance between monomers along the Z‐axis is ca 3.35 Å, while the intermolecular center‐to‐center distance between two neighboring benzene rings in the stack is ca 3.70 Å. Many intermolecular C−H⋅⋅⋅O short contacts (about 2.2 Å apart) between the glycol chains help stabilize the aggregates. The central bipyridine units in a stack are involved in aromatic (π–π) interactions and in weak intermolecular C−H⋅⋅⋅N hydrogen bonding (*d*_C‐H⋅⋅⋅N_ ≈3.52 Å). The heats of formation (Δ*H*_f_) obtained from the PM6 calculations were used to estimate Δ*H*_f_ (in kJ mol^−1^) for the following aggregation processes: 1+1→2 (+66), 2+1→3 (−353), and 3+1→4 (−340). The initial aggregation step seems to be a non‐spontaneous seed formation (dimerization), followed by more spontaneous elongation steps, namely trimer and tetramer formation, which would fit with a cooperative supramolecular growth. For the initial nucleus formation (1+1→2), it can be assumed that the monomer activation predominantly constitutes a preorganization step, which includes a decrease in flexibility of the freely rotatable bipyridine center accompanied by a planarization of the OPE cores. For instance, the torsion angle involving the central rotatable C−C bond and both the nitrogen atoms in the same monomer unit slightly increases from 177° to 179° upon dimerization. With the more ordered, planar structure of the nucleus, initial contact to another monomer (i.e. 2+1→3) is facilitated and sufficiently strong interactions provided. These essential changes require loss of conformational freedom, turning the activation into an unfavorable step, as indeed predicted from the PM6 calculations.

From all the above observations, an aggregation model for **1** can be proposed, as shown in Scheme 1 and Figure S14. At high temperatures (≈353 K), **1** exists in a molecularly dissolved state. Slow cooling initiates the self‐assembly, which is mainly driven by aromatic and hydrophobic interactions in aqueous media. Such close intermolecular interactions between the aromatic scaffolds explain the increase in absorbance upon cooling. Furthermore, the redshift of the absorption band upon aggregation suggests a slightly twisted molecular arrangement, as predicted by solid‐state NMR studies, most likely to minimize the steric bulkiness posed by the glycol chains of adjacent molecules. The relatively flexible nature of the glycol chains allows the creation of a hydrophilic shell protecting the conjugated OPE and bipyridine units from the aqueous environment. However, we hypothesize that not all aromatic fragments are efficiently shielded from the surrounding media, which drives the individual fibers to further organize laterally into dense clusters, as suggested by TEM and absorption studies. These superstructures might be further stabilized by the interdigitation of the glycol chains of neighboring monomers mediated by water molecules.^19^

### Impact of acid on the supramolecular polymerization of 1: *cis*‐locked 1‐H^+^

2,2’‐bipyridines in a monomeric state are well‐known for their sensitivity towards various acids,6a leading to the formation of the monoprotonated bipyridine species, even in the presence of a large excess of acid.^6^ The monoprotonated bipyridine adduct is favored over the bis‐protonated species since the former benefits from intramolecular stabilization by N‐H^+^⋅⋅⋅N interactions (Scheme 1).^6^ Consequently, the protonation of 2,2’‐bipyridine is accompanied by a geometrical change from the more extended *trans*‐ to the V‐shaped *cis*‐conformation (Scheme 1). Despite that this conformational change anticipates important implications at the supramolecular level, it is surprising that the pH‐responsive behavior of bipyridines has been almost exclusively investigated in a monomeric state in “good” solvents. Thus, we sought to investigate whether and to what extent the protonation of **1** to yield **1‐H^+^** would influence the supramolecular properties of the system.

To this end, we initially investigated the responsiveness of ligand **1** to the addition of trifluoroacetic acid (TFA) in aqueous solution by UV/Vis titration studies. For these experiments, equivalent conditions as those used for previous temperature‐dependent studies of **1** were used (1.5×10^−5^ m, 298 K). The corresponding absorption spectrum of **1** prior to acid addition clearly indicates a highly aggregated state. Subsequently, increasing aliquots of a diluted solution of TFA (water/TFA=3:1) were added gradually until the pH reached approximately 1.1 and the titration was monitored by UV/Vis absorption spectroscopy (Figure S15). Surprisingly, only a marginal decrease in absorption without any additional spectral changes is observed upon TFA addition, even if large amounts of acid are added (ca. 200 equivalents) and high concentration is used (Figure S15). These results indicate that the aqueous assemblies of **1** negligibly respond towards TFA under the investigated experimental conditions. Proof of that is the absence of a clear redshifted band that is characteristic for the monoprotonated form of bipyridine.6e We hypothesize that the dense molecular packing of the π‐backbone induced by strong aggregation most likely blocks the access of the surrounding protons in acidic media and consequently prevents the protonation of the bipyridine units. This phenomenon is most likely reinforced by the strong solvation and shielding effect of the peripheral glycol chains in aqueous media. The minor decrease in absorption upon TFA addition can be explained by slight changes in the hydrogen bonding structure between the outer hydrophilic shell of the fibers and surrounding water molecules at low pH, which slightly reduce the aggregate solubility. Notably, the absorption band of **1** in aqueous medium remains unchanged over time upon TFA addition under highly acidic conditions (pH≈1), which emphasizes a remarkable stability of the assemblies (Figure S15).

**Figure 4:**
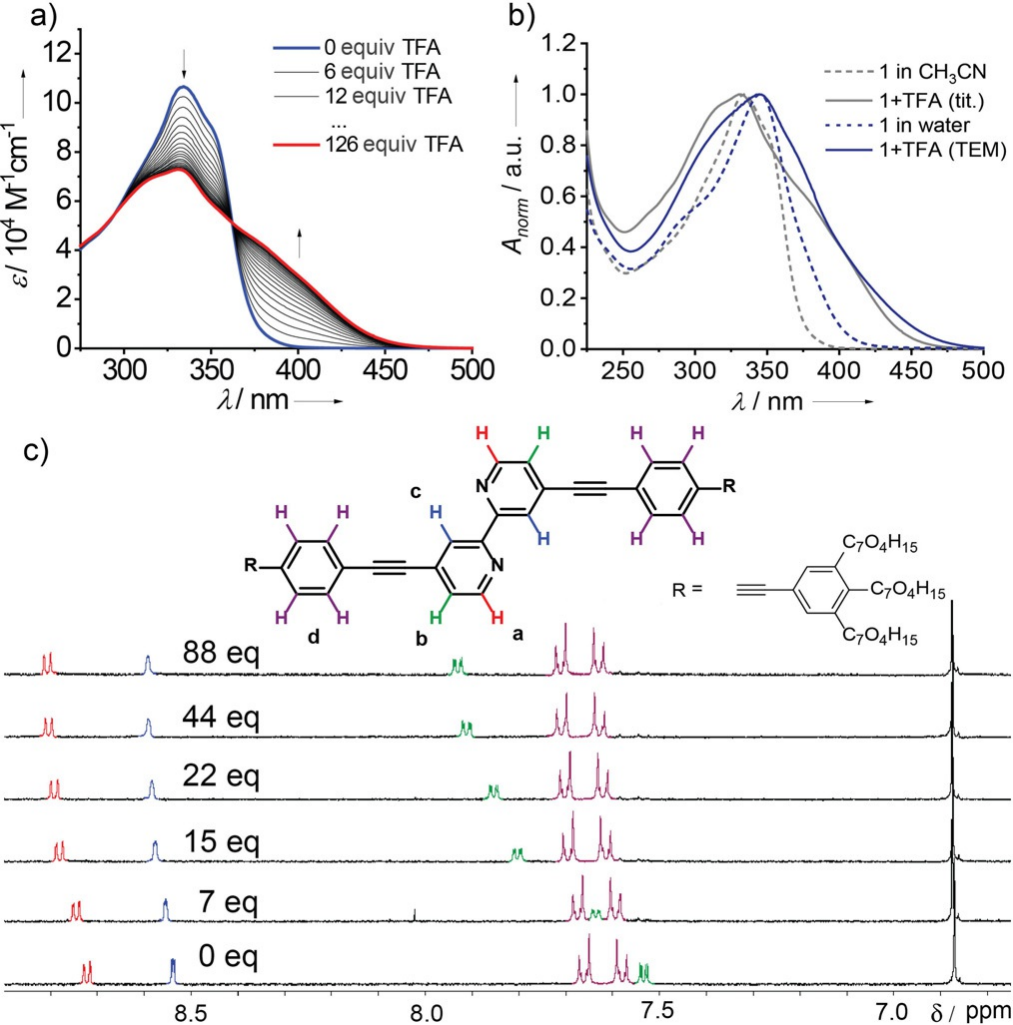
a) UV/Vis titration studies of **1** in acetonitrile (1.5×10^−5^ m) using TFA from 0 up to ≈126 equivalents. Arrows indicate the spectral changes upon addition of acid. b) Spectral differences between pure **1** (dotted lines) and its protonated form **1‐H^+^** (solid lines) in acetonitrile (grey) and water (blue). c) ^1^H NMR titration studies of **1** in CD_3_CN (1.4×10^−4^ m) upon addition of 0, 7, 15, 22, 44 and 88 equivalents TFA (CF_3_COOD) from bottom to top.

Given that the aqueous assemblies of **1** as such do not respond to acid, another experimental setup was required to allow the protonation of the bipyridine unit. Solvents such as acetonitrile (MeCN) and THF were chosen for protonation studies of **1** considering the good solubility of both the ligand and TFA in these media and their miscibility with water. Additionally, **1** exists in a monomeric state in both MeCN and THF, which should facilitate the exposure of the bipyridine moieties to the acid molecules. For the titration experiments, a solution **1** in MeCN (1.4×10^−5^ m) and a highly diluted TFA solution (TFA/MeCN=1:49) were prepared. Addition of TFA in steps of approximately 6 equivalents caused remarkable changes in the absorption spectrum, as shown in Figure 4 a. The absorption band at 334 nm showed a gradual decrease with the concomitant formation of an intense shoulder at around 400 nm up to ≈126 equivalents, after which no significant spectral changes were observed. This newly formed transition can be assigned to the monoprotonated form of the bipyridine unit of **1**. Subsequently, deprotonation studies were carried out using an organic base 1.8‐diazabicyclo[5.4.0]undec‐7‐en (DBU) (Figure S16). The addition of the diluted base (DBU/MeCN=1:24) resulted in complete and reversible recovery of the spectral features of the neutral (uncharged) ligand **1** (Figure S16). The emergence of a single isosbestic point during protonation and deprotonation studies indicate a conformational switch between two distinct species in equilibrium: the *trans*‐ and *cis*‐ states (**1** and **1‐H^+^**) (Scheme 1). The redshifted band in absorption upon protonation can be rationalized by the formation of an intramolecular N−H⋅⋅⋅N hydrogen bridge that causes an increased planarization and, consequently, π‐conjugation of the aromatic surface of **1**.^6^

To complement the UV/Vis studies, the protonation process of **1** was also followed by ^1^H NMR titration experiments in CD_3_CN at a concentration of 1.4×10^−4^ m. To monitor the protonation in small steps, the concentrated TFA (CF_3_COOD) was diluted using CD_3_CN (1:19). Seven samples with varying amount of TFA were prepared, ranging from 0 to 133 equivalents. As no NMR shifts occur beyond 88 equivalents, the spectrum with 133 equivalents TFA is not shown in Figure 4 c. The ^1^H NMR spectrum of **1** in CD_3_CN (Figure 4 c, bottom spectrum) exhibits sharp signals, suggestive of a molecularly dissolved state. Upon addition of acid, the aromatic proton signals H_a‐d_ undergo a downfield shift to a different extent whereas those corresponding to the oligoethylene chains remain unaffected (Figure S17). More specifically, the signals for H_a_, H_b_ and H_c_ are significantly shifted downfield with the incremental addition of acid (Figure 4 c), being this effect most pronounced for the proton signals H_b_. The effect on the remaining proton signals of the aromatic core (H_d_ and H_e_) is rather insignificant, considering that these protons are located far from the protonated bipyridine unit. Proof of this is the fact that the proton signal H_d_ undergoes a moderate downfield shift, while the most distant protons H_e_ of the terminal phenylene ring (black peaks in Figure 4 c) are completely unaffected. Comparing the TFA titration experiments of **1** in water and „good“ organic solvents, we can conclude that the formation of the monoprotonated species **1‐H^+^** can only be monitored in detail in the latter media, whereas strong aggregation in water prevents the TFA molecules to penetrate the acid‐responsive bipyridine units.

Since the direct addition of TFA to aggregated **1** in water/THF (99:1) did not entail a significant effect, an alternative preparation method should be introduced to investigate the effect of bipyridine protonation on the self‐assembly. To this end, a small volume of a pale yellow‐colored solution of **1** at 4.8×10^−3^ m in THF was initially prepared, where **1** exists in a monomeric state. To this solution, a large excess of concentrated TFA was added to ensure full protonation of the bipyridine unit, causing an immediate intensification of the yellow color. Subsequently, a small amount of this solution was diluted with a large excess of water to reach a concentration of 7.1×10^−4^ m and the sample was kept at room temperature in a vial with perforated cap for a few weeks to allow for equilibration and evaporation of the traces of THF. The resulting aqueous solution was then investigated by UV/Vis absorption measurements. Figure 4 b shows the comparison of the absorption spectra of **1** and its corresponding mono‐protonated form **1‐H^+^** in a monomeric (in MeCN) and in an aggregated state (water). In order to better identify the differences, all spectra have been normalized. As previously observed, neutral **1** undergoes a redshift and slight spectral broadening upon aggregation when moving from MeCN to water. Similarly, the UV/Vis spectrum of **1‐H^+^** also shows a redshift upon aqueous self‐assembly compared to the monomer spectrum in MeCN. Simultaneous to this redshift, the absorption band of aggregated **1‐H^+^** further broadens up to 500 nm. In particular, the monoprotonated form **1‐H^+^** exhibits a characteristic shoulder at 375 nm, which is appreciable both in the monomeric (MeCN) and aggregated state (water). This suggests that the new preparation method (creation of **1‐H^+^** in THF, followed by addition of water and removal of THF) leads to an aggregate spectrum that significantly differs ‐it is further redshifted‐ from the aggregate spectrum of the neutral species **1**.

**Figure 5:**
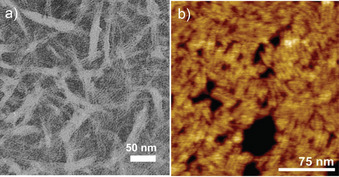
a) TEM and b) AFM images of self‐assembled **1‐H^+^** (*c*=7×10^−4^ m) prepared in water onto a copper‐coated copper grid and mica, respectively.

In analogy to **1**, we also attempted to gain mechanistic insights into the aqueous self‐assembly process of **1‐H^+^** by UV/Vis studies. However, temperature‐dependent experiments are unfortunately not applicable under such highly acidic conditions due to the precipitation of the sample upon heating (Figure S18). Therefore, the denaturation (disassembly) of aggregated **1‐H^+^** was monitored spectroscopically by gradual addition of aliquots of monomeric **1** in an acidic solution of THF/TFA (2×10^−5^ m, 1 m THF solution, 0.1 m aqueous solution, *T*=25 °C). Pleasingly, a transition from self‐assembled **1‐H^+^** to the characteristic monomeric absorption spectrum of **1‐H^+^** *via* an isosbestic point could be identified upon optimization of the experimental conditions of water/THF ratio and amount of TFA (Figure S19 a). Plotting the spectral changes at different wavelengths *vs*. the volume fraction of acidic THF leads to a denaturation curve that cannot be described by the isodesmic or cooperative mechanisms (Figure S19 b–d). In particular, the appreciation of a two‐step curve seems to indicate that **1‐H^+^** might follow an anti‐cooperative mechanism, as recently observed for amphiphilic OPE‐based Pt^II^ complexes.18b On this basis, a different aggregate morphology is expected for the aqueous solutions of **1** and **1‐H^+^**. In order to find this out, microscopy investigations were conducted for the same aggregate solution used for UV/Vis studies. TEM on carbon‐coated copper grid revealed one‐dimensional assemblies of **1‐H^+^** that show a strong tendency to agglomeration (Figure 5 a and S20). Remarkably, the length of the assemblies upon protonation is considerably smaller than that of the neutral ligand **1**. The TEM studies were further complemented by atomic force microscopy (AFM) on mica surface (Figures 5 b and S21). AFM provided a higher resolution than TEM and therefore allowed the visualization of agglomerated, individual rods with a diameter of 3.2±0.3 nm and a length of up to 70 nm. These structures further self‐assemble into densely packed regions on the hydrophilic substrate.

### Quantum chemical calculations of 1‐H^+^

To gain insights on the packing of **1‐H^+^**, dispersion‐corrected PM6 calculations of the *cis*‐locked species were performed using the MOPAC package, where ground‐state geometries of monomer and dimer in vacuum were optimized (Scheme 1 and Figure S22). The proton attached to the pyridine helps stabilize the *cis* form via intramolecular H‐bonds to the other pyridine nitrogen (*d*(N−H⋅⋅⋅N)=2.3 Å), while the repulsion between the beta‐hydrogens of both pyridines (2.3 Å apart) hampers the planarization of the ligand, the torsion angle involving N‐C‐C‐N being 23 degrees. The two individual OPE scaffolds themselves adopt a planar conjugated arrangement. The average distance from one nitrogen to the last oxygen of the corresponding glycol chain is about 2 nm, which, together with the fiber diameter of around 3 nm obtained from AFM and TEM suggest a zigzag, antiparallel packing of the monomer units of **1‐H^+^** inside the supramolecular fiber. In this arrangement, subsequent monomer units are rotated relatively to each other by 180 degrees (see dimer of **1‐H^+^** in Scheme 1), which helps minimize unfavorable repulsions between the positively charged bipyridine‐H^+^ units. This arrangement also renders fibers with a vanishingly small dipole moment, which is generally preferred by molecules inside supramolecules.^20^

**Figure 6:**
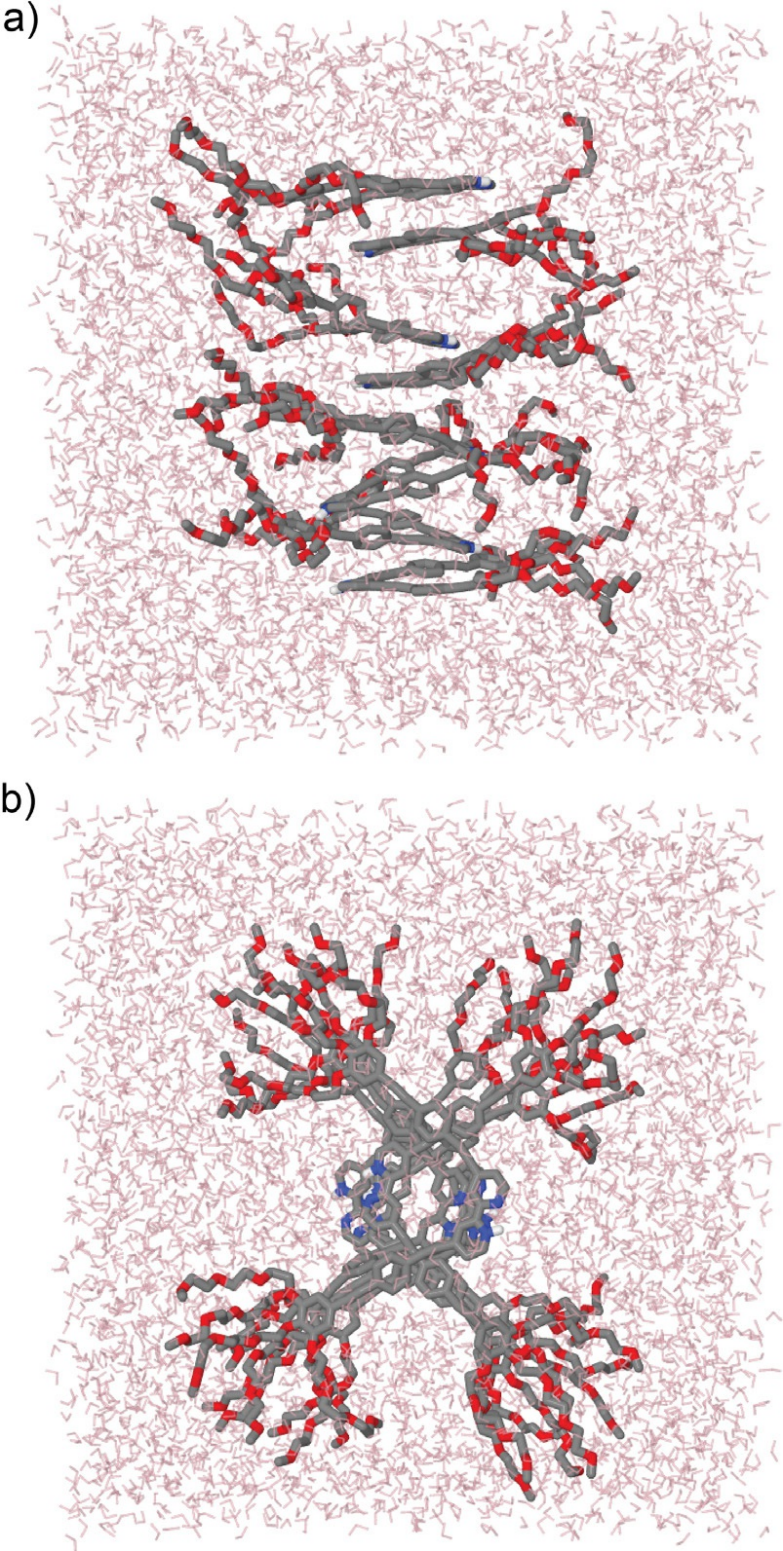
Snapshots of a 1 ns‐long MD simulation (NPT, 298 K, 1 atm) of an octamer of compound **1**‐**H+** in water. Nonpolar hydrogens were omitted for clarity. Color code: C(gray), N(blue), O(red), H(white), water molecules=pink.

Classical molecular dynamics (MD) simulations were performed to further investigate this zigzag aggregation using the Tinker program and MMFF94 force field. The simulation box was comprised of an octamer of **1‐H^+^** surrounded by 5142 (explicit) water molecules. After initial geometry optimization and equilibration, the system was simulated using the NPT ensemble at 298 K and 1 atm for 1 ns (Figure 6, further details of the simulations are described in the Supporting Information). The zigzag arrangement was indeed stable in water throughout the whole simulation, although monomer units exhibit some degree of mobility inside the fiber due to the well‐known dynamic nature of supramolecular systems. The middle OPE rings of neighboring molecules were in close contact to stabilize the stacks by attractive aromatic interactions, supported by hydrophobic forces. Simultaneously, the flexible glycol chains created a hydrophilic shell around the zigzag stacks to minimize the unfavorable contact of the hydrophobic scaffold to the surrounding aqueous media. The glycol chains of every third monomer unit were interacting with each other *via* weak intermolecular CH⋅⋅⋅O short contacts. Such type of interactions involving glycol chains have been previously observed to greatly contribute to the stabilization of aqueous assemblies of metal‐based amphiphilic π‐systems.18a On average, the distance between oxygens from the end of the glycol chains of two consecutive monomer units within the fiber was about 3.0 nm, which is in good agreement with the AFM/TEM measurements already discussed. The suggested zigzag antiparallel packing model is one of the possible arrangements how the monomer units of **1‐H^+^** can assemble into a thin 1D fiber, as shown by microscopy studies. In this arrangement, the positively charged bipyridine‐H^+^ units would be alternately oriented to opposite sides to minimize electrostatic repulsion and at the same time, the polar TEG chains would shield a great part of the supramolecular column. Most likely, the increasingly higher repulsion of charged groups upon fiber growth is responsible for the termination of the supramolecular growth explaining why the 1D fibers upon of **1‐H^+^** have a limited length. Therefore, the acid‐responsive bipyridine conformational switch can be efficiently exploited to tune the aggregate growth.

## Conclusions

In summary, we have demonstrated the use of 2,2’‐bipyridines as acid‐responsive conformational switches in supramolecular polymerization. To that end, we have designed a bipyridine‐based bolaamphiphilic derivative **1** where the 2,2’‐bipyridine core has been substituted at the 4,4’‐positions with OPE fragments decorated with polar TEG chains. Under standard conditions, the bipyridine unit of **1** primarily exists in its *trans*‐conformation, leading to a linear arrangement of the molecular aromatic core. This high degree of preorganization enables a cooperative supramolecular polymerization of **1** into well‐ordered bundled fibers when water is used as aggregation‐inducing solvent. The robustness of these supramolecular structures in water is reflected in their high stability upon addition of an acid (TFA), which is attributed to strong hydrophobic and aromatic interactions of the aromatic core and the shielding effect of the peripheral TEG chains. In contrast, monoprotonation of the 2,2’‐bipyridine core to generate **1‐H^+^** can occur efficiently if TFA is added to **1** in a molecularly dissolved state in organic solvents. Interestingly, addition of water to the monomeric solution of the monoprotonated form **1‐H^+^** induces supramolecular polymerization, although to a much lower extent than neutral species **1**. This attenuated growth of **1‐H^+^** into shorter fibers may be possibly attributed to the destabilization of larger aggregates due to the positively charged monomer units as well as the decreased tendency of the positively charged monomers to associate due to better stabilization in polar media. We envisage that the use of bipyridines as acid‐sensitive units to tune self‐assembly is expected to open up new directions in the field of conformational switches and stimuli‐responsive systems.

## Conflict of interest

The authors declare no conflict of interest.

## Biographical Information

*Gustavo Fernández received his PhD in 2009 from the Universidad Complutense de Madrid (Spain) under the supervision of Profs. Nazario Martin and Luis Sanchez for work on donor–acceptor systems based on C_60_. He then joined the group of Prof. Frank Würthner at the University of Würzburg (Germany) as a Humboldt postdoctoral researcher working on merocyanine dye aggregates. Between December 2010 and September 2015, he headed an independent junior research group in the same institute focusing on the self‐assembly and self‐sorting of* π*‐systems. Since September 2015, he is Professor of Organic and Supramolecular Chemistry at the University of Münster (Germany), where he heads a research group working on the self‐assembly and stimuli‐responsive behavior of functional BODIPY dyes and metal‐containing* π‐*systems*.

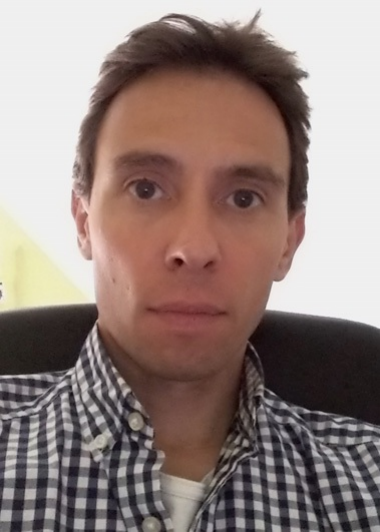

## Supporting information

As a service to our authors and readers, this journal provides supporting information supplied by the authors. Such materials are peer reviewed and may be re‐organized for online delivery, but are not copy‐edited or typeset. Technical support issues arising from supporting information (other than missing files) should be addressed to the authors.

Supplementary
