# Supplementary material for: Tuning Aqueous Supramolecular Polymerization by an Acid‐Responsive Conformational Switch
Source: Chemistry. 2020 Jul 14;26(44):10005–13. doi: 10.1002/chem.202001566 (PMC7496824; doi:10.1002/chem.202001566)
Supplement: Supplementary file 1 — Supplementary [file CHEM-26-10005-s001.pdf]

Chemistry–A European Journal

Supporting Information

Tuning Aqueous Supramolecular Polymerization by an Acid-Responsive Conformational Switch

Christina Rest,[a] Divya Susan Philips,[b] Torsten Dünnebacke,[b] Papri Sutar,[b] Angel Sampedro,[b] Jörn Droste,[c] Vladimir Stepanenko,[a] Michael Ryan Hansen,*[c] Rodrigo Q. Albuquerque,*[b] and Gustavo Fernández*[b]

Index

A. Materials and Methods **S3**

General

NMR measurements

Variable-temperature DOSY NMR experiments

Mass spectrometry

UV-Vis absorption and fluorescence spectroscopy

Fluorescence quantum yield measurements

Transmission electron microscopy (TEM) experiments

Atomic force microscopy (AFM) experiments

Denaturation experiments

Quantum chemical calculations

B. Synthesis and Characterization.. S**7**

C. Supplementary Figures and Tables... S**14**

UV/Vis spectroscopy

Fluorescence spectroscopy

Fluorescence quantum yield

Thermodynamic parameters

NMR spectroscopy

Solid sate NMR

Transmission electron microscopy

Atomic force microscopy

Optimized geometry

D. References S**26**

A. Materials and Methods

General: All solvents were dried according to standard procedures. Reagents were used as purchased. All air-sensitive reactions were carried out under argon or nitrogen atmosphere. Flash chromatography was performed using silica gel (Merck Silica 60, particle size 0.04–0.063 nm).

NMR measurements: ^{1}H and ^{13}C NMR spectra were recorded on a *Bruker Avance 400* (^{1}H: 400 MHz; ^{13}C: 100.6 MHz) and a *Bruker AV300* (^{1}H: 300 MHz; ^{13}C: 100.6 MHz). Deviating temperature is explicitly mentioned when used. The recorded spectra were referenced to the remaining resonance signals of the deuterated solvents ($CDCl_3$: 7.26 ppm (^{1}H); DCM: 5.32 ppm (^{1}H); DMSO: 2.50 ppm (^{1}H,); THF: 1.73 ppm (^{1}H); D_2O: 4.79 (^{1}H); MeOD: 4.87 ppm (^{1}H)). The coupling constant *J* of the measured spin multiplets is given in Hertz (Hz) and the chemical shifts *δ* are given in reference to the chemical shift of trimethylsilane (0 ppm). The abbreviations used to analyze the recorded spectra are: *s* (singlet), *d* (doublet), *t* (triplet), *q* (quartet), *m* (multiplet).

Solid-state ^{13}C{^{1}H} CP/MAS NMR measurements were done on a Bruker AVANCE III 300 spectrometer operating at 7.05 T using a 4.0 mm MAS WVT H/X double-resonance probe and a Bruker DSX 400 spectrometer operating at 9.4 T using 4.0 mm MAS BVT H/X/Y triple-resonance probe. The ^{1}H MAS NMR experiments were conducted on a Bruker DSX 500 spectrometer operating at 11.74 T using 2.5 mm MAS DVT H/X/Y triple-resonance probe. All samples were packed into ZrO_2 rotors with either 2.5 mm or 4.0 mm o.d. The 2.5 mm rotors were sealed with Vespel© top-caps and bottom-caps. All spectra were recorded at ambient conditions with adamantane as external reference for determining the radio-frequency field strengths and referencing the chemical shift scale (δ(^{1}H) = 1.85 ppm and δ(^{13}C) = 29.47 ppm).[S1] The ^{1}H MAS NMR and 2D ^{1}H-^{1}H DQ-SQ correlation spectra were recorded with a 90° pulse length of 2.7 µs. The 2D ^{1}H-^{1}H DQ-SQ correlation spectra were recorded with Back-to-Back (BaBa) DQ recoupling using the xy16 phase cycle.[S2] The ^{13}C{^{1}H] CP/MAS NMR spectra were recorded with a proton 90° pulse length of 4.0 µs using a ramp from 70-100 % on the ^{13}C channel for a fixed radio-frequency field strength on ^{13}C. The 2D ^{13}C{^{1}H} FSLG-LGCP HETCOR NMR spectra were recorded with 100 kHz FSLG[S3] decoupling during the proton chemical shift evolution time and the ^{1}H chemical shift scale was scaled with a factor of 0.578. The CP transfer was achieved by employing a 70-100 % ramp on the ^{13}C channel fulfilling the LGCP condition. All ^{13}C-detected MAS NMR spectra were acquired with ^{1}H decoupling using the SW_f-TPPM15 decoupling scheme.[S4] Data analysis, processing, and plotting was done using the Bruker Topspin 4.0.6 software, dmfit[S5] and Python 3.7.

For ^{13}C and ^{1}H signal assignment of the solid-state NMR spectra, gas-phase DFT calculations of isolated molecules were conducted with the TURBOMOLE.[S6] A coarse geometry optimization with the meta-GGA functional TPSS[S7] followed by an optimisation with def2-TZVP[S8] was done for the *cis* and *trans* isomer of the bipyridine group, respectively. The SCF loop was converged at an E_H of 10^{-7}. The basis sets were taken from the EMSL data base. The isotropic ^{13}C and ^{1}H chemical shieldings were calculated from the geometry-optimized structures using the hybrid functional B3LYP.[S9] Equation 1 and 2 below were used to convert the calculated ^{13}C and ^{1}H chemical shieldings into chemical shifts.

$$\delta_{\mathrm{iso}}(^{13}\mathrm{C}) = -0.8839 \cdot \sigma_{\mathrm{C}} + 171.47 \quad (1)$$

$$\delta_{\mathrm{iso}}(^{1}\mathrm{H}) = -0.929 \cdot \sigma_{\mathrm{H}} + 29.837 \quad (2)$$

Equation 1 and 2 were obtained by a correlating known ^{13}C and ^{1}H chemical shifts for a range of small model compounds (e.g. Pyridine, Benzene, Furan) with the corresponding DFT-calculated chemical shieldings.

Variable-temperature diffusion ordered spectroscopy (DOSY) NMR experiments: Variable-temperature DOSY NMR experiments at 5.0 x 10^{-3} M in ACN-d_3/D_2O (1:1) were performed to monitor the variation of the hydrodynamic radius during aggregation. The increased concentration in the sample of **1** compared to UV/Vis and emission is required to obtain sufficiently resolved signals for the NMR experiment. Also, if aggregates of **1** are investigated by NMR in pure water, no signals can be identified due to strong aggregation, making necessary the use of a co-solvent (acetonitrile). In order to decrease the tendency to self-assemble and avoid precipitation, the percentage of acetonitrile was optimized to a final ratio of 1:1. As shown in Figure S5, the solutions in the NMR tubes between ca 297 K and 283 K remain clear, suggesting the presence of monomeric **1** under these conditions. However, an increase of the turbidity of the sample is appreciable between 283 K and 280 K (Figure S5), indicating a transition from the monomer to the aggregate state. By NMR, however, there is a sudden drop in the diffusion coefficient already when cooling below 298 K. The hydrodynamic radius can be calculated applying the Stokes-Einstein equation, obtaining values of 12 Å for the monomer and 22 Å for the aggregate (Figure S6). Further cooling of the sample caused a critical broadening of the signals, suggesting that the calculated hydrodynamic radius corresponds to the nucleation step, where still signals can be identified. Assuming an average length for the molecule of 4.4 nm, the diameter can be calculated applying a rod-shaped model to be 1.3 nm and 3.3 nm respectively for the monomer and nucleating aggregate species.[S10] Due to the precipitation of the sample below 280 K, monitoring of the hydrodynamic radius during the elongation step was not possible.

Mass spectrometry: HR-ESI mass spectra were measured on a Bruker MicroTOF system. MALDI spectra were recorded using an autoflex II instrument (Bruker) and DCTB as matrix.

UV-Vis absorption and fluorescence spectroscopy: UV/Vis absorption spectra were recorded using a JASCO-V770 or JASCO-V750 spectrophotometer with a spectral bandwidth of 1.0 nm and a scan rate of 400 nm min^{-1}. Variable temperature measurements were performed with a ramp rate of 0.1 K min^{-1} unless otherwise specified. For all measurements, spectroscopic grade solvents were used. All experiments were carried out using quartz cuvettes with optical paths of 1 cm or 1 mm. Fluorescence spectra were recorded on a Jasco FP-8500 spectrofluorimeter equipped with the same water circulation unit.

Fluorescence quantum yield measurements: To quantify the emission of compound **1** in different solvents, the fluorescence quantum yield was determined (Table S1). As reference compound, quinine sulphate in 0.05 M H_2SO_4 (refractive index $\eta = 1.33$) was chosen whose quantum yield is defined as 0.52. To yield an absorption value <0.05 a concentration of 4.0 - 5.0 x 10^{-7} M was used.

TEM experiments: To reveal the architecture of the self-assembled aggregates of compound **1**, aggregate samples were investigated by TEM. To facilitate sample preparation, the compound was initially dissolved in a small volume of THF and diluted to the required concentration by addition of a large excess of water. Subsequently, this solution was kept for a few days/weeks in a closed vial with perforated cap to allow for equilibration and ensure the evaporation of the traces of THF. The corresponding aqueous solutions of **1** at 5.7 x 10^{-4} M and 8.0 x 10^{-4} M were subsequently drop-casted onto a carbon-coated copper grid (Carbon Type B (15-25 nm) on 200 mesh, with Formvar; Ted Pella, Inc.). The solutions were allowed

to evaporate under ambient conditions. The dried specimen was studied using a Siemens Elmiskop 101 Electron Microscope with an accelerating voltage of 80 kV. The data were analyzed using Digital Micrograph software. For the TEM investigations of **$1\text{-}H^+$,** a similar preparation protocol as for the neutral ligand **1** was followed. Initially, **1** was dissolved in a small volume of THF. Next, this solution was treated with a large excess of concentrated TFA (~280 eq.) to ensure protonation of the bipyridine moiety yielding **$1\text{-}H^+$**. Finally, a large volume of water was added to this solution to induce aggregation and the sample was kept at room temperature in a vial with perforated cap for a few weeks to allow for equilibration and evaporation of the traces of THF. The resulting aqueous solution (7.1×10^{-4} M) was then investigated by TEM in an identical way as for **1**.

AFM experiments: AFM images were recorded on a Multimode® 8 SPM System (AXS Bruker). Silicon cantilevers with a nominal spring constant of 9 Nm^{-1} and with resonant frequency of ~150 kHz and a typical tip radius of 7 nm (OMCL-AC200TS, Olympus) were employed. The aqueous solution of **$1\text{-}H^+$** (7.1×10^{-4} M) was spin-coated onto mica at room temperature. The solvent was evaporated under vacuum before the measurement.

Denaturation experiments: Denaturation experiment of **1** (2×10^{-5} M in water) was performed by gradual addition of increasing amounts of an equally concentrated solution of molecularly dissolved **1** in THF. Thus, addition of molecularly dissolved **1** in THF to an aggregate solution of **1** in water at the same concentration leads to a gradual disassembly of the aggregates. Upon increasing the volume fraction of THF, the λ_{max} undergoes a blue shift (347 to 337 nm), while the shoulder at around 380 nm vanishes. The overall spectroscopic changes reveal an isosbestic point at 365 nm and are in good agreement with the spectroscopic properties observed in the temperature-dependent experiments in pure water. However, unlike the experiments in pure water (Figure 1c), λ_{max} at 337 nm shows an increase in absorption upon disassembly in denaturation studies. This may be explained by the use of different solvent mixtures in both experiments (up to 50% THF in water for denaturation, pure water for temperature-dependent experiments), which heavily influences the overall extinction coefficient ε (see solvent dependent UV-Vis spectra in Figure 1a). By plotting the evolution of α_{Agg} as a function of the volume fraction of THF (Figure S4) the aggregation process can be successfully fitted to the denaturation model by *Meijer and co-workers.*[S11] revealing a Gibbs free energy of $\Delta G^0 = -52.4$ kJ·mol^{-1} and a cooperativity parameter of $\sigma = 0.07$.

We also performed the denaturation experiments of **$1\text{-}H^+$**. However, in contrast to neutral **1**, these experiments can only be performed under a rather limited set of experimental conditions, as otherwise deprotonation of **$1\text{-}H^+$** upon disassembling the sample, or precipitation under highly acidic conditions takes place. In order to avoid deprotonation of **$1\text{-}H^+$** in neutral solvents, acidic solutions of TFA in water (0.1 M) and THF (1.0 M) were used to further investigate the underlying aggregation process. Due to the insolubility of **$1\text{-}H^+$** in more acidic aqueous solutions (e.g. 1 M), a significantly lower concentration had to be chosen for this solvating medium. Additionally, a certain volume of THF (a minimum of 3%) in water is required to avoid precipitation of the protonated molecule. With the aim to obtain a stable yet non-precipitated aggregate solution of **$1\text{-}H^+$** in water, initially a small volume (100 µL) of a higher concentrated acidic solution in THF (1 M, $c = 5.9 \cdot 10^{-4}$ M) was injected into a solution of TFA in water (0.1 M, 2900 µL) under vigorous stirring. Initially, increasing volume fractions of **$1\text{-}H^+$** in acidic THF were added to this solution. However, during the initial additions of **$1\text{-}H^+$** in acidic THF to the aggregate solution of **$1\text{-}H^+$** in acidic water, fluctuations in the absorbance without a clear trend are observed, which might be the result of partial de- protonation/reprotonation processes of **$1\text{-}H^+$** that compete with the expected disassembly upon addition of the good solvent. Thus, the THF content was raised to 10% for the initial measurement. The denaturation process (2 x 10$^-$

^{5} M) can be subsequently monitored by UV(Vis spectroscopy in a range of volume fractions of THF between 0.1 (10%) and 0.23 (23%) (Figure S19), as indicated by the sharpening of the transition bands with a simultaneous increase of the overall absorption. Upon addition of around 22%, the characteristic absorption spectrum of monomeric **1-H^{+}** is observed (see comparison with Figure 4b). Further addition of **1-H^{+}** in THF above 24% leads to phase separation, preventing the accessibility of a stable plateau. The slightly different aggregate spectrum for **1-H^{+}** when comparing Figure 4b and S19 (the spectrum shown in Figure 4b is slightly red-shifted) is attributed to the higher amount of THF and lack of aging used in denaturation studies. For the associated mechanistic analysis, the evolution of α_{Agg} was plotted as a function of the volume fraction of THF for three different wavelengths (323 nm (λ_{max} aggregate), 333 nm (λ_{max} monomer) and 340 nm). For comparison, the resulting data points were attempted to be fitted according to the isodesmic (red line) and cooperative (green line) aggregation model, respectively (Figure S19).

Quantum chemical calculations: Semiempirical quantum chemical calculations at the dispersion-corrected PM6 level were carried out to optimize the geometries of stacks of compound **1**. The calculations were performed in vacuum using the MOPAC package. The reliability of the optimized structures was checked by confirming the absence of imaginary vibrational frequencies in the predicted vibrational spectra. The monomer charge and multiplicity used in the calculations were 0 and 1 (=singlet), respectively.

The MD simulations were carried out using the Tinker package and MMFF94 force field. Partial charges were assigned to all structures from previously optimized ones at the semiempirical PM6 level. Electrostatic interactions were computed using the Particle-Mesh-Ewald (PME) technique. Van der Waals interactions were calculated using the buffered 14-7 functional (cutoff = 1.1 nm), which takes into account dispersion interactions. The simulations were done using the NPT ensemble at 298 K and 1 atm, and Periodic Boundary Conditions (PBC). The simulation box (about 5 nm of side) was filled with 8 protonated monomers and 5142 water molecules with bond lengths constrained by the rattle algorithm. The equations of motion were integrated by the Verlet scheme with timestep of 1 fs for a total simulation time of 1 ns. MD snapshots were visualized with the VMD package.

B. Synthesis and Characterization

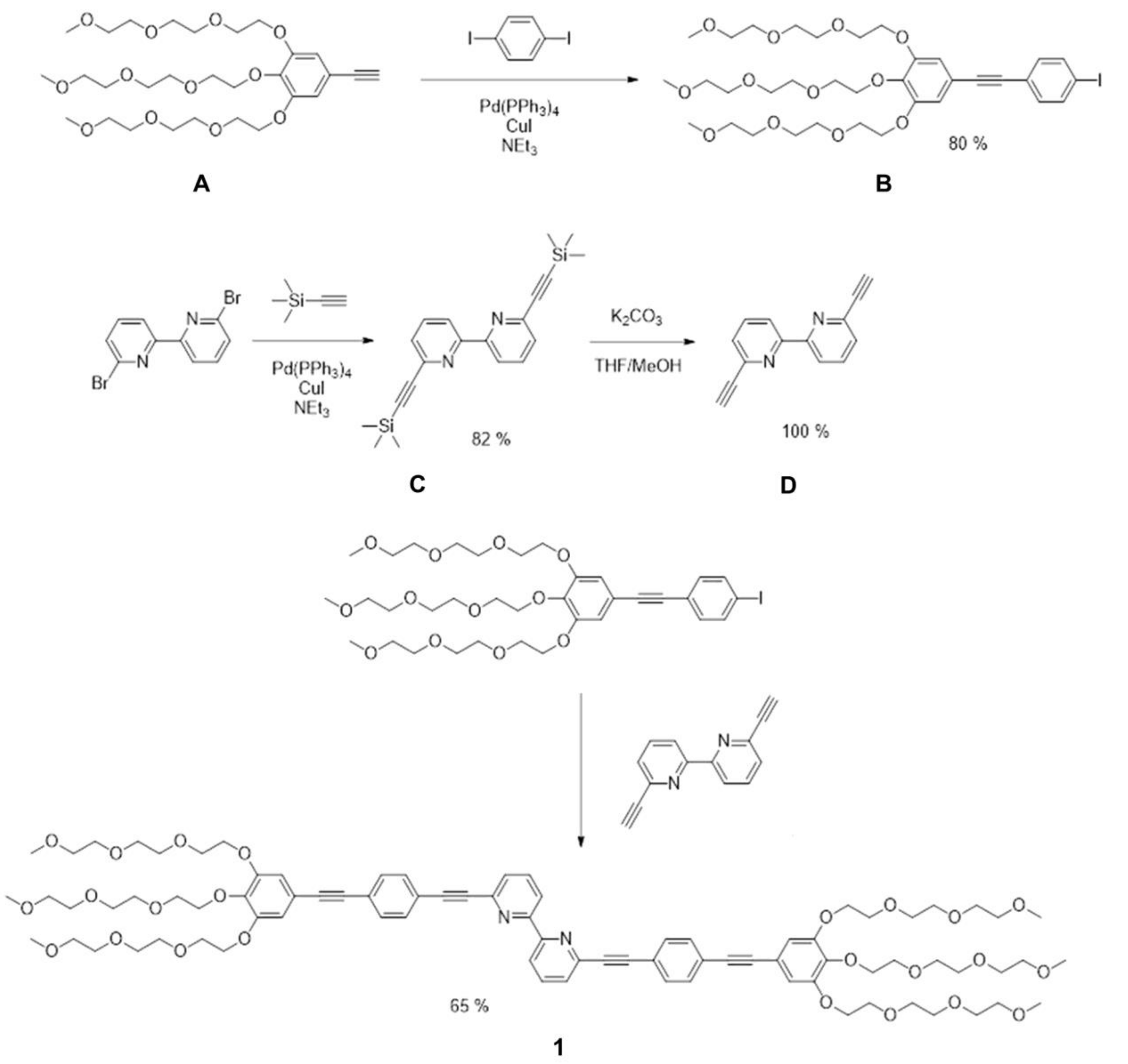

Scheme S1: Synthesis of **1**.

5-ethynyl-1,2,3-tris(2-(2-(2-methoxyethoxy)ethoxy)ethoxy)-benzene (**A**)[S12] was prepared following reported synthetic procedures and showed identical spectroscopic properties. 5-((4-iodophenyl)ethynyl)-1,2,3-tris(2-(2-(2-methoxyethoxy)ethoxy)ethoxy)benzene (**B**)[S13], 4,4'-bis((trimethylsilyl)ethynyl)-2,2'-bipyridine (**C**)[S12] and 4,4'-diethynyl-2,2'-bipyridine (**D**)[S14] were synthesized by modifying previously reported procedures.

Synthesis of 5-((4-iodophenyl)ethynyl)-1,2,3-tris(2-(2-(2-methoxyethoxy)ethoxy)ethoxy)benzene (B)

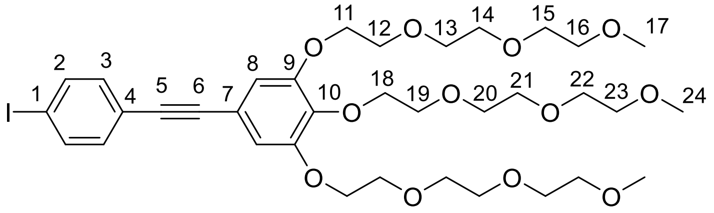

$C_{35}H_{51}IO_{12}$
790,2425 g/mol

1,4-Diiodobenzene (4,11 g; 12,44 mmol; 3.00 eq), $Pd(PPh_3)_4$ (239.2 mg; 5 mol%) and CuI (79.0 mg; 10 mol%) were dispersed in freshly distilled and degassed NEt_3 (60 mL) and stirred at room temperature for 20 min. Next, a solution of 5-ethynyl-1,2,3-tris(2-(2-(2-methoxyethoxy)ethoxy)ethoxy)-benzene (**A**) (2.44 g; 4.15 mmol; 1 eq) in freshly distilled THF (4 mL) was added dropwise and the reaction mixture was heated under stirring at 60 °C for 62.5 h. After cooling to room temperature, the mixture was diluted with EtOAc, filtered over Celite® and the solvents were removed under vacuum. The residue was purified by column chromatography (SiO_2; 1 % to 3 % MeOH in DCM). The product obtained was a brownish, highly viscous liquid (2.61 g; 3.30 mmol; 80 %).

HRMS (ESI, Micro Tof, MeOH): *m/z* = found: 813.2324 (100) $[M+Na]^+$, calc. for: $C_{35}H_{51}IO_{12}Na^+$ 813.2323.

^{1}H NMR ($CDCl_3$, 600 MHz, 293 K): δ (ppm) = 7.68 (*d*; *J* = 8.5 Hz; 2H; H_2), 7.23 (*d*; *J* = 8.5 Hz; 2H; H_3), 6.76 (*s*; 2H; H_8), 4.19 – 4.15 (*m*; 6H; H_{11}+H_{18}), 3.85 (*t*; *J* = 5.0 Hz, 4H, H_{12}), 3.79 (*t*; *J* = 5.0 Hz; 2H; H_{19}), 3.75 – 3.70 (*m*; 6H; H_{13}+H_{20}), 3.68 – 3.62 (*m*; 12H; H_{14}+H_{15}+H_{21}+H_{22}), 3.56 – 3.52 (*m*; 6H; H_{16}+H_{23}), 3.37 (*s*; 3H; H_{24}), 3.37 (*s*; 6H; H_{17}).

^{13}C NMR ($CDCl_3$, 151 MHz, 293K): δ (ppm) = 152.7 (C_9), 139.7 (C_{10}), 137.7 (C_2), 133.2 (C_3), 122.9 (C_4), 117.8 (C_7), 111.4 (C_8), 94.2 (C_1), 90.9 (C_6), 87.7 (C_5), 72.6 (C_{18}), 72.1 (C_{23}), 72.1

(C_{16}), 71.0 (C_{13}), 70.9 (C_n), 70.8 (C_{21}), 70.7 (C_{15}+C_{22}), 70.7 (C_{19}), 70.7 (C_{20}), 69.8 (C_{12}), 69.1 (C_{11}), 59.2 (C_{17}), 59,2 (C_{24}).

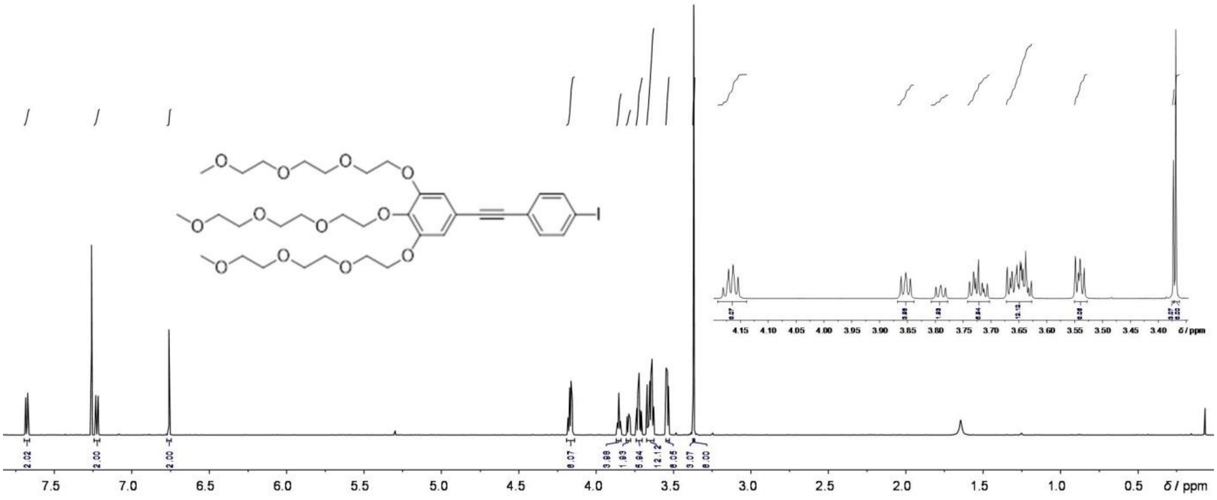

^{1}H NMR ($CDCl_3$, 600 MHz, 299 K) of **B**.

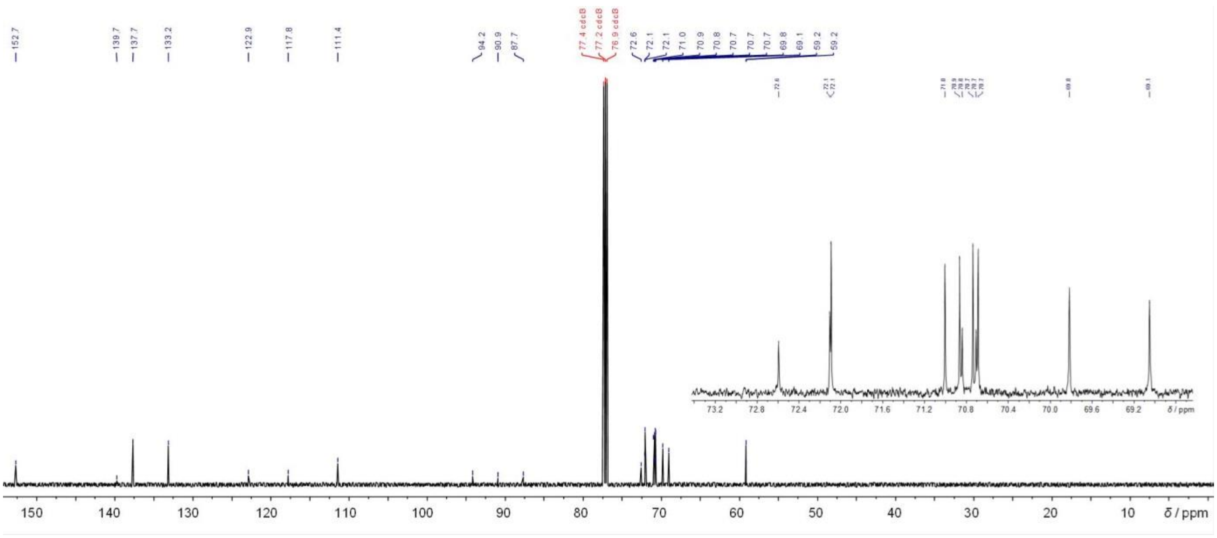

^{13}C NMR ($CDCl_3$, 150 MHz, 299 K) of **B**.

Synthesis of 4,4'-bis((trimethylsilyl)ethynyl)-2,2'-bipyridine (C)

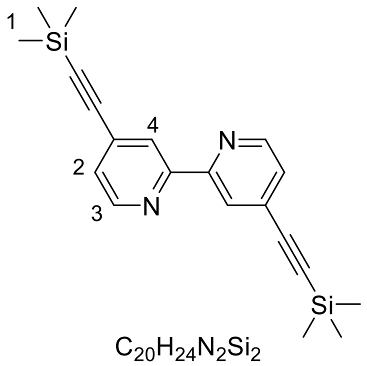

$C_{20}H_{24}N_2Si_2$
348,1478

4,4’-Dibromo-2,2’-bipyridine (941.9 mg, 3.00 mmol, 1 eq), $Pd(PPh_3)_4$ (104.0 mg, 3 mol%) and CuI (51.4 mg, 9 mol%) were dissolved in freshly distilled and degassed NEt_3 (25 mL) and stirred at room temperature for 20 min. Next, trimethylsilylacetylene (1.650 g, 2.33 mL, 5.6 eq) was added dropwise and the reaction mixture was stirred at 40 °C for 10 min, then heated up to 80 °C and stirred at this temperature for 20.5 h. All volatile compounds were removed under reduced pressure and the residue was purified by column chromatography (SiO_2, 1 % MeOH/DCM to 5 % MeOH/DCM). The residue was dissolved in pentane and filtered over a thin layer of SiO_2. The product was obtained as a colorless solid (857.3 mg, 2.46 mmol, 82 %).

HRMS (ESI, Micro Tof, MeOH): *m/z* = found: 349.1566 (88) $[M+H]^+$, 371.1384 (37) $[M+Na]^+$, 719.2854 (100) $[2M+Na]^+$, calc. for: $C_{20}H_{25}N_2Si_2^+$ 349.1551, $C_{20}H_{24}N_2Si_2Na^+$ 371.1376, $(C_{20}H_{24}N_2Si_2)_2Na^+$ 719.2854.

^{1}H NMR ($CDCl_3$, 300 MHz, 293 K): δ (ppm) = 8.61 (*d*, J = 5.0 Hz, 2H, H_3), 8.46 – 8.39 (*m*, 2H, H_4), 7.31 (*dd*, J = 5.0, 1.6 Hz, 2H, H_2), 0.26 (*s*, 18H, H_1); $\boldsymbol{R_f}$ = 0.19 (2.5 % MeOH/DCM).

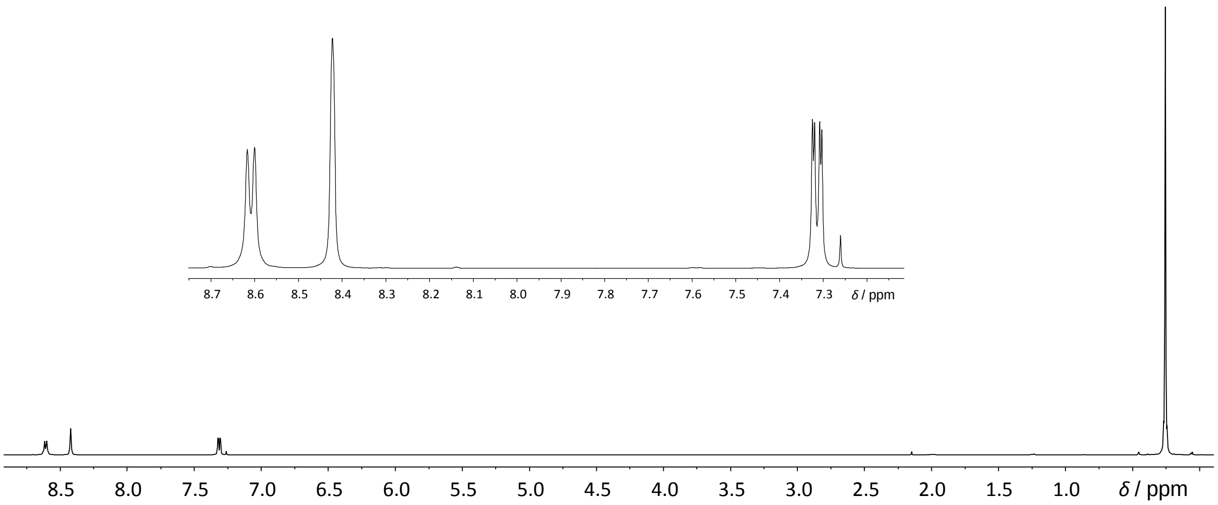

^{1}H NMR ($CDCl_3$, 300 MHz, 293 K) of 4,4'-bis((trimethylsilyl)ethynyl)-2,2'-bipyridine (**C**).

Synthesis of 4,4'-diethynyl-2,2'-bipyridine (D)

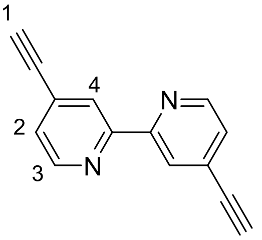

$C_{14}H_8N_2$
204,0687

C (208.9 mg, 0.6 mmol, 1 eq) and K_2CO_3 (663.4 mg) were dissolved in a mixture of MeOH and THF (1:1, 3 mL) and the reaction mixture was stirred at room temperature for 3 h. The solvents were removed under reduced pressure and the residue was dissolved in DCM (25 mL) and water (25 mL). The phases were separated and the organic layer was washed with water (2x20 mL, 1x30 mL) and dried over $MgSO_4$. All volatile compounds were removed under reduced pressure and the product was obtained as a pale yellow solid without further purification (124.0 mg, 0.6 mmol, 100 %)

HRMS (ESI, Micro Tof, MeOH): *m/z* = found: 205.0765 (100) $[M+H]^+$, 227.0586 (100) $[M+Na]^+$, 431.1266 (100) $[2M+Na]^+$, calc. for: $C_{14}H_9N_8^+$ 205.0766, $C_{14}H_8N_2Na^+$ 227.0580, $(C_{14}H_8N_2)_2Na$ 431.1273.

^{1}H NMR ($CDCl_3$, 400 MHz, 293 K): δ (ppm) = 8.66 (*s br.*, 2H, H_3), 8.49 (*s*, 2H, H_4), 7.38 (*dd*, *J* = 5.0, 1.6 Hz, 2H, H_2), 3.32 (*s*, 2H, H_1).

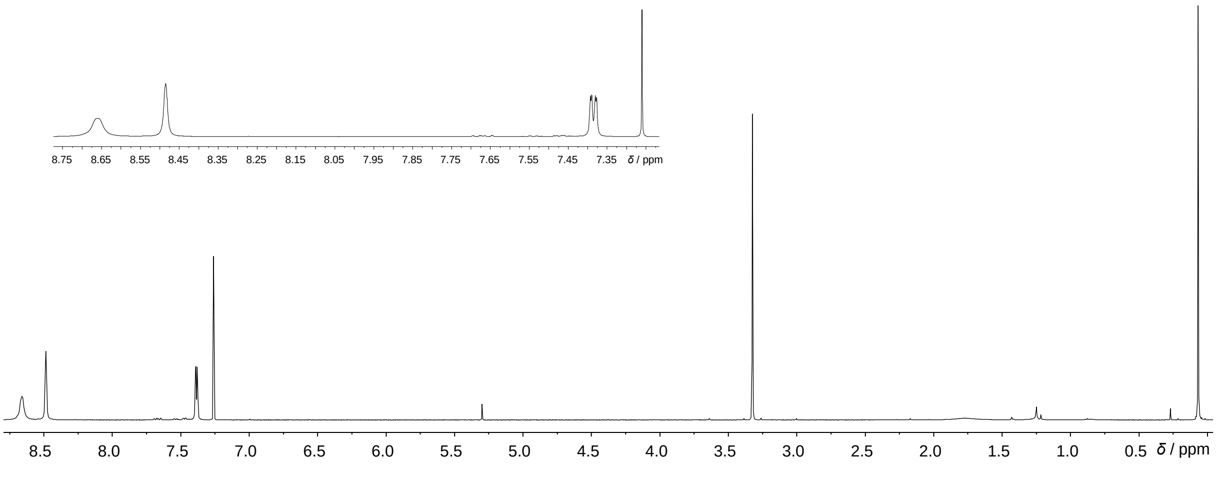

^{1}H NMR ($CDCl_3$, 400 MHz, 293 K) of 4,4'-diethynyl-2,2'-bipyridine (**D**).

Synthesis of 4,4'-bis((4-((3,4,5-tris(2-(2-(2-methoxyethoxy)ethoxy)ethoxy)phenyl)ethynyl)phenyl) ethynyl)-2,2'-bipyridine (1)

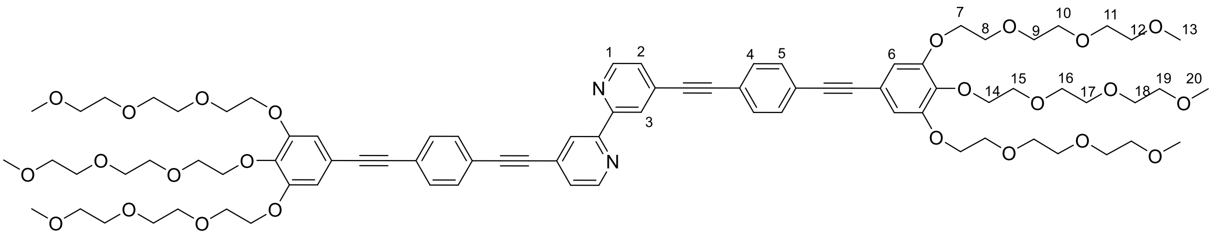

$C_{84}H_{108}N_2O_{24}$
1528,7292

B (558.3 mg, 0.7 mmol, 2 eq), $Pd(PPh_3)_4$ (24.3 mg, 6 mol%) and CuI (0.3 mg, 4 mol%) were combined in freshly distilled and degassed NEt_3 (7 mL) and the reaction mixture was stirred at room temperature for 20 min. **D** (72.1 mg, 0.35 mmol, 1 eq) was added and the reaction mixture was stirred at 60 °C for 21 h. All volatile compounds were removed under reduced pressure and the residue was purified by column chromatography (SiO_2, 2.5 % MeOH/DCM). The desired product was obtained as a yellow paste (357.4 mg, 0.23 mmol, 65 %).

MALDI-TOF: *m/z*: calculated for $[C_{84}H_{108}N_2O_{24}Na]^+$ $[M+Na]^+$:1551.718; found: 1551.709.

HRMS (ESI, pos. Mode, $CH_3CN/CHCl_3$ (1:1)): m/z: calculated for $[C_{84}H_{108}N_2O_{24}Na]^+$ $[M+Na]^+$: 1551.7184; found: 1551.7190.

Elemental analysis: Calculated for $C_{84}H_{108}N_2O_{24}$: C, 65.95; H, 7.12; N,1.83; found (++ V_2O_5): C, 65.60; H, 7.37; N, 1.89

^{1}H-NMR ($CDCl_3$, 500 MHz, 293 K): δ (ppm) = 8.69 (*dd*, *J* = 5.0, 0.9 Hz, 2H, H_1), 8.53 (*m*, 2H,H_3), 7.55 – 7.50 (*m*, 8H, H_4+H_5), 7.42 (*dd*, *J* = 5.0, 1.6 Hz, 2H, H_2), 6.78 (*s*, 4H, H_6), 4.21 – 4.14 (*m*, 12H, H_7+H_{14}), 3.86 (*t*, *J* = 4.9 Hz, 8H, H_8), 3.80 (*t*, *J* = 5.0 Hz, 4H, H_{15}), 3.77 – 3.70 (*m*, 12H, H_9+H_{16}), 3.70 – 3.60 (*m*, 24H, H_{10}+H_{17}+H_{11}+H_{18}), 3.59 – 3.51 (*m*, 12H, H_{12}+H_{19}), 3.37 (*s*, 6H, H_{20}), 3.37 (*s*, 12H, H_{13}).

^{13}C NMR ($CDCl_3$, 126 MHz, 293 K): δ (ppm) = 155.74, 152.65, 149.38, 139.74, 132.49, 132.00, 131.69, 125.63, 124.24, 123.40, 121.95, 117.77, 111.46, 93.88, 91.95, 88.79, 88.15, 77.41, 77.16, 76.91, 72.58, 72.06, 72.05, 70.96, 70.82, 70.79, 70.69, 70.68, 70.65, 70.64, 70.59, 70.43, 69.79, 69.02, 59.13, 59.12.

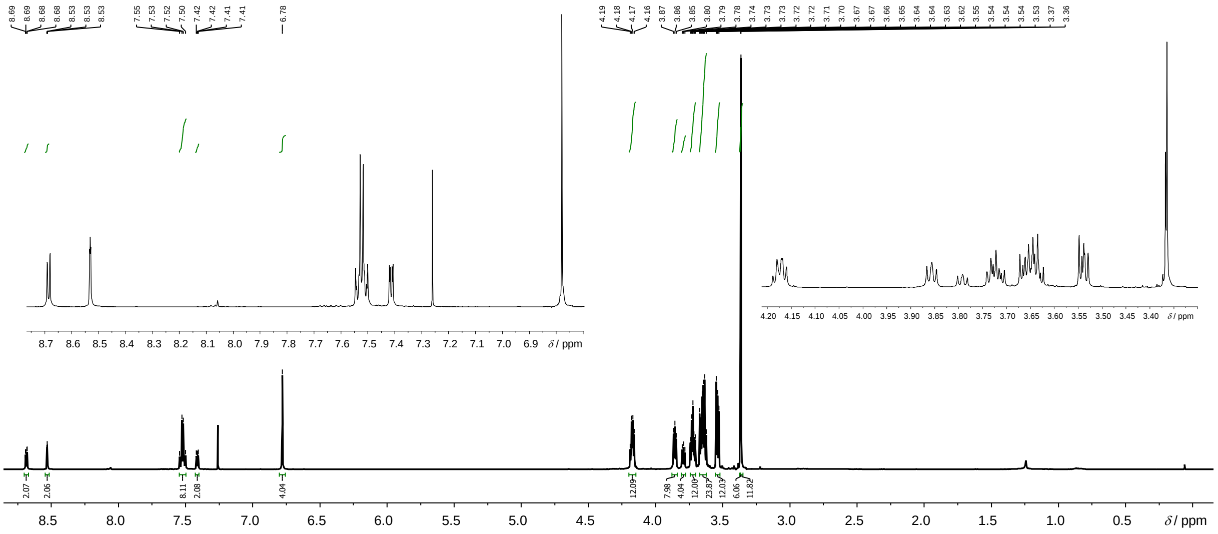

^{1}H NMR ($CDCl_3$, 500 MHz, 293 K) of **1**

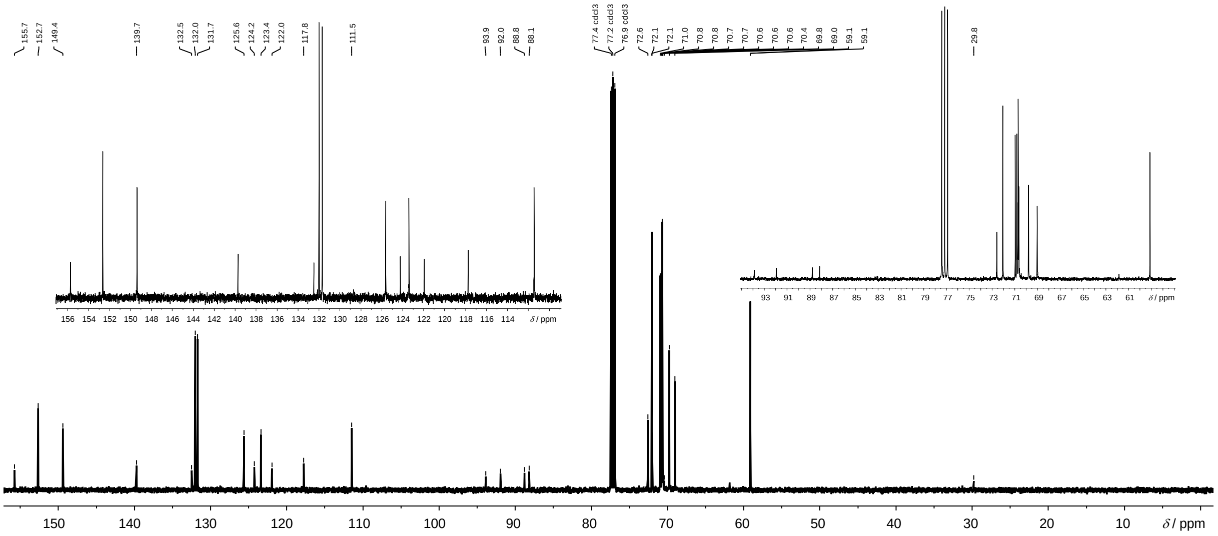

^{13}C NMR ($CDCl_3$, 126 MHz, 293 K) of **1**

C. Supporting Figures and Tables

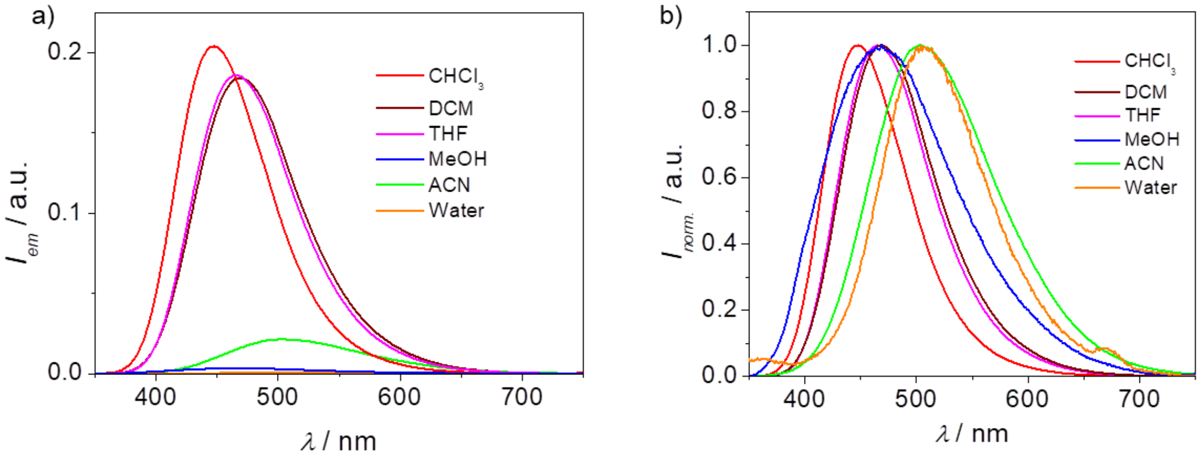

Figure S1: a) Solvent-dependent fluorescence spectra of **1** (0.9 -1.2 x 10^{-5} M) at room temperature (λ_{ex} = 335 nm) and b) the corresponding normalized spectra to understand the solvatochromic behavior of **1**.

Table S1: Fluorescence quantum yield ϕ_{FL} for **1** in different solvents at 4.0 - 5.0 x 10^{-7} M.

Solvents	**$CHCl_3$**	**THF**	**CH_2Cl_2**	**CH_3CN**	**MeOH**	**H_2O**
ϕ_{FL}	0.52±0.05	0.52±0.04	0.50±0.04	0.08±0.005	0.01±0.001	0.01±0.002

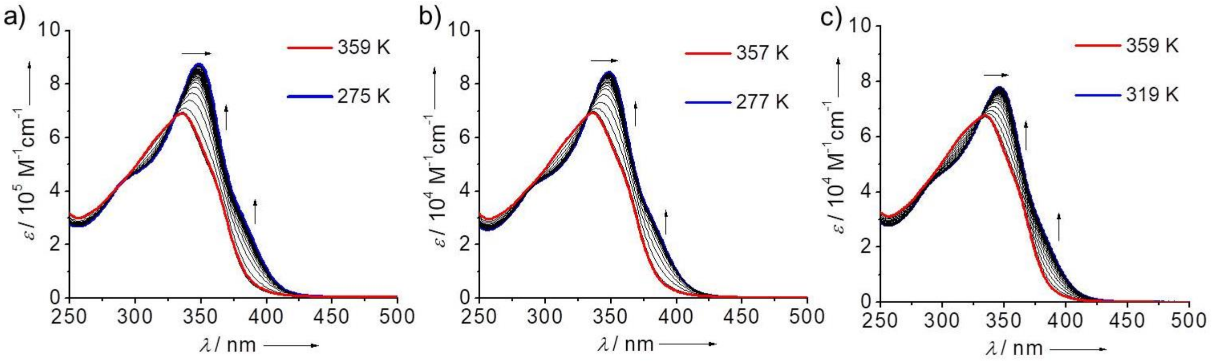

***Figure S2**:* Temperature-dependent UV/Vis experiments of **1** in a) water (2.9 x 10^{-5} M) upon decreasing temperature from 359 K to 275 K with 1 K/min; b) in water/THF = 99:1 (2.4 x 10^{-5} M) upon decreasing temperature from 357 K to 277 K with 1 K/min; c) in water/THF = 99:1 (1.5 x 10^{-5} M) upon decreasing temperature from 359 K to 319 K with a cooling rate of 0.1 K/min.

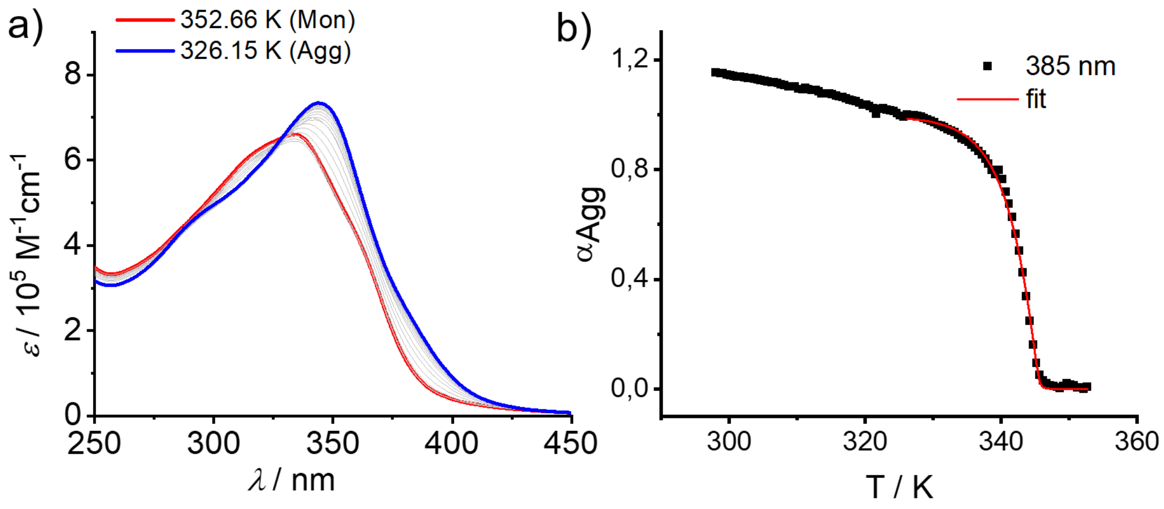

***Figure S3**:* a) Temperature-dependent UV/Vis absorption spectra of **1** in water (2.0 x 10^{-5} M) upon cooling from 353 to 326 K with 0.1 K/min. b) Cooling curve obtained by monitoring absorption changes at 385 nm and their successful fitting to the cooperative self-assembly model (red line). The data points below ca. 326 K continuously increase after having reached the aggregation plateau, which has been attributed to fiber bundling.[S15] These data points below 326 K have not been considered for the cooperative fit.

Table S2: Thermodynamic parameters associated with the supramolecular polymerization of **1** in water as obtained by fitting the degree of aggregation α_{Agg} as a function of T according to the K-K_2 model.[S16, S17]

ΔH^0 / kJ·mol^{-1}	$\Delta H^0_{\mathrm{nucl}}$ / kJ·mol^{-1}	ΔS^0 /kJ·mol^{-1} ·K^{-1}	ΔG_{298} /kJ·mol^{-1}	T_e / K	K_{nucl} / L·mol^{-1}	K_{el} / L·mol^{-1}	σ / 10^{-4}
-262.35	-20.32	-0.67	-62.69	345.13	42.06	50034.36	8.41

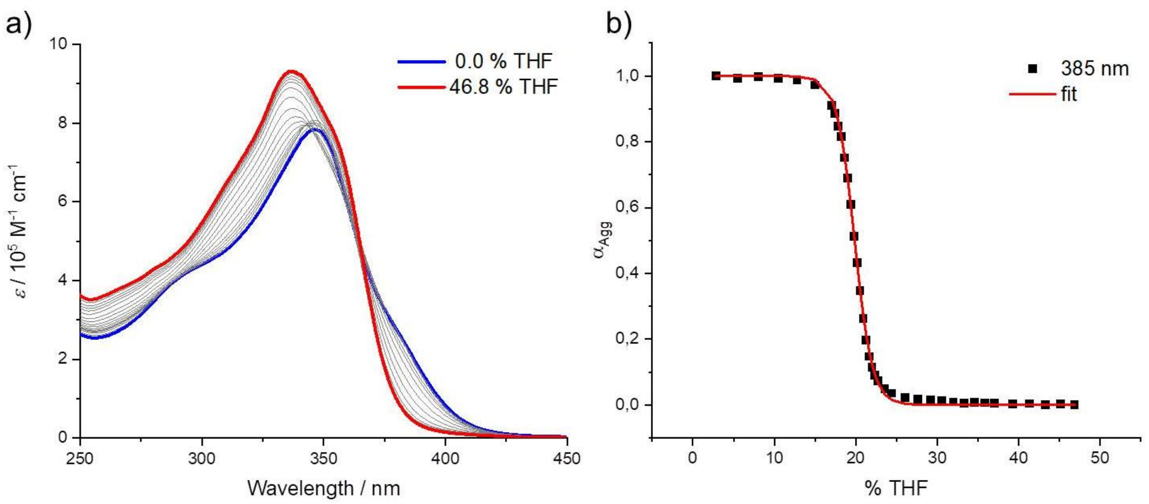

***Figure S4**:* a) UV-vis spectra of **1** in water (c = 2 x 10^{-5} M, aggregated state, blue plot) and subsequent denaturation upon gradual addition of **1** in a good solvent (THF, c = 2 x 10^{-5} M, monomeric state, red spectrum). b) Denaturation of the aggregates showing the plot of the degree of aggregation (α) against the amount of good solvent (THF) and cooperative fit.

Table S3: Thermodynamic parameters extracted from denaturation studies of **1** as obtained by fitting the degree of aggregation α_{Agg} as a function of the THF volume fraction according to the denaturation model.[S18]

ΔG^0 / kJ·mol^{-1}	*m* / kJ·mol^{-1}	σ
-52.4±3.16	122.0±16.4	0.070±0.065

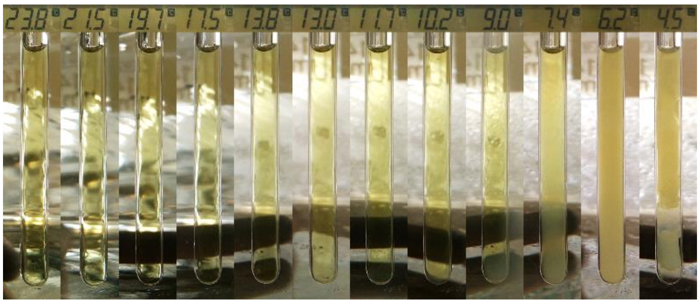

Figure S5: Sequence of photographs of **1** dissolved in ACN-d_3/D_2O (1:1) at a concentration of 5.0 x 10^{-3} M. One equivalent of Et_3N was added to the sample in order to maintain the pH of the solution slightly basic, ensuring the bipyridine moiety is not protonated, and also as internal NMR reference. The sample was slowly cooled by addition of small amounts of ice to the stirred water bath. Temperature, indicated on top of each picture in °C, was monitored with a digital thermoprobe.

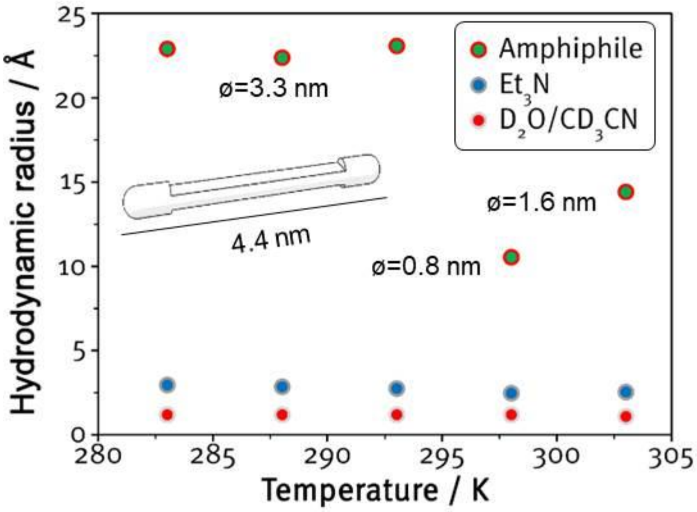

Figure S6: The sample shown in the previous picture (**1**, 5.0 x 10^{-3} M, ACN-d_3/D2O (1:1), 1 equiv. Et_3N) was analyzed by DOSY NMR experiments at different temperatures. From the diffusion coefficients obtained for all species present, the hydrodynamic radii are calculated using the Stokes-Einstein equation. The calculated value for the signals corresponding to the solvents is averaged to 1.2 Å, as expected due to the smaller size of these molecules. For Et_3N, the hydrodynamic radius resulted in 2.7 Å, which is in agreement with a Stokes radius of 3.4 Å. The difference observed may be attributed to the lack of accuracy of the technique in order to determine the diffusion coefficient for small molecules. The fact that the radii observed remains virtually constant independently of the temperature is coherent, as these molecules are not affected by the aggregation of **1**. The diameters indicated for **1** are the results of applying a rod-shaped model, considering a molecular length of 4.4 nm.

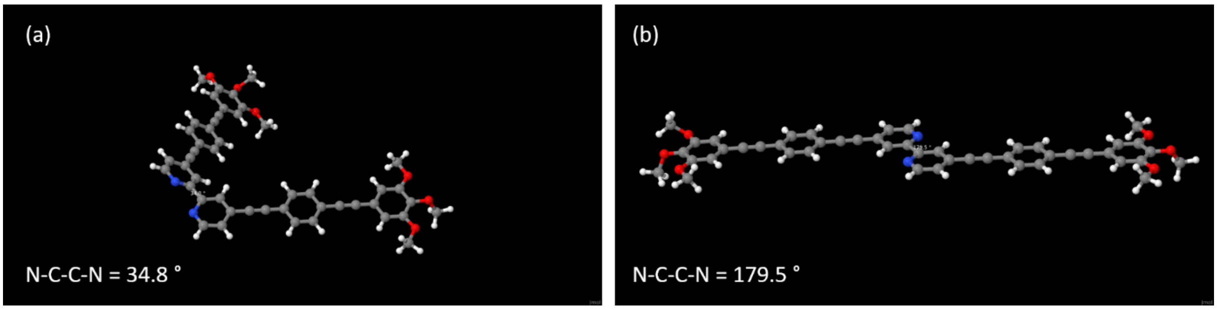

Figure S7: TZVP optimized structure used for the chemical shift calculations of **1** in (a) *cis* and (b) *trans* conformation. The ethylene glycol chains were substituted by methoxy groups.

Table S4: Experimental and calculated chemical shifts for the bipyridine atoms affected by conformational change. For C-atom labelling, see the chemical structure below in Figure S8.

atom	**δ_{exp}(^{13}C) / ppm**	**$\delta_{calc,\ trans}$ (^{13}C) & $\delta_{calc,\ trans}$ (^{1}H) / ppm**		**$\delta_{calc,\ cis}$ (^{13}C) & $\delta_{calc,\ cis}$ (^{1}H) / ppm**	
1	153/155	155		157	
2	122/123/125/127	124	8.9	126	7.8
3	125/127	133		133	
4	114/117	125	7.2	123	7.2
5	148/149	147	8.6	149	8.7
6	90-96	97		96	
7	90-96	100		100	
8	122	123/127		123/127	
9	132/133	131/133	7.5	131/133	7.5
10	90-96	94		94	
11	90-96	101		101	
12	118	120/121		120/121	
13	110	108-111 & 121[a]	6.7	108-111 & 121[a]	6.7
14	152	154/155		154/155	
15	138	140/141		141/145	

[a] This carbon atom is sensitive to the conformation of the oxygen of the adjacent methoxy group and is only present in the calculation.

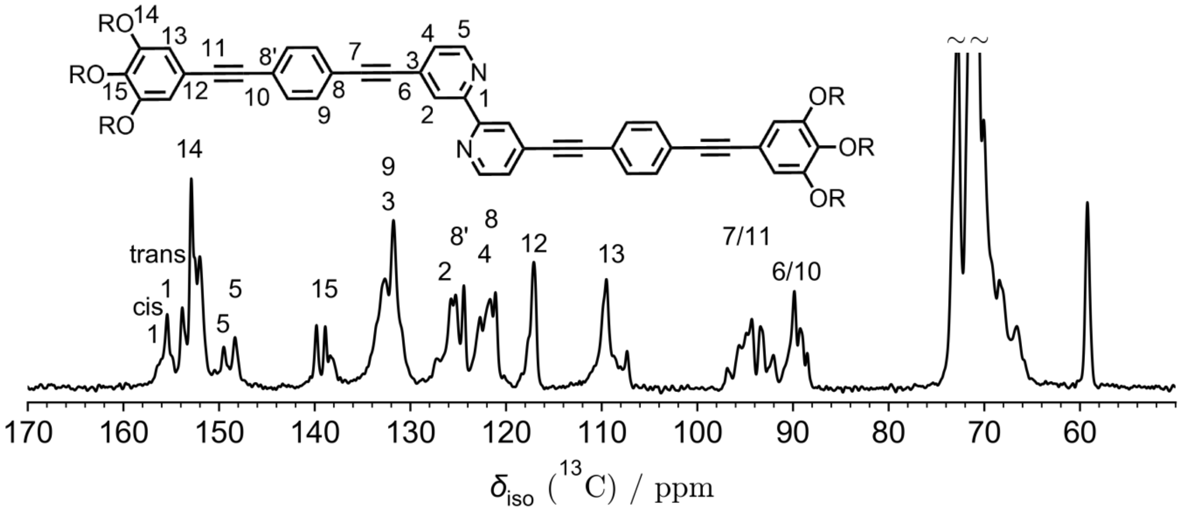

Figure S8: Solid-state $^{13}C\{^1H\}$ CP/MAS NMR spectrum of **1** recorded at 9.4 T employing a MAS frequency of 11.0 kHz and a CP time of 3 ms.

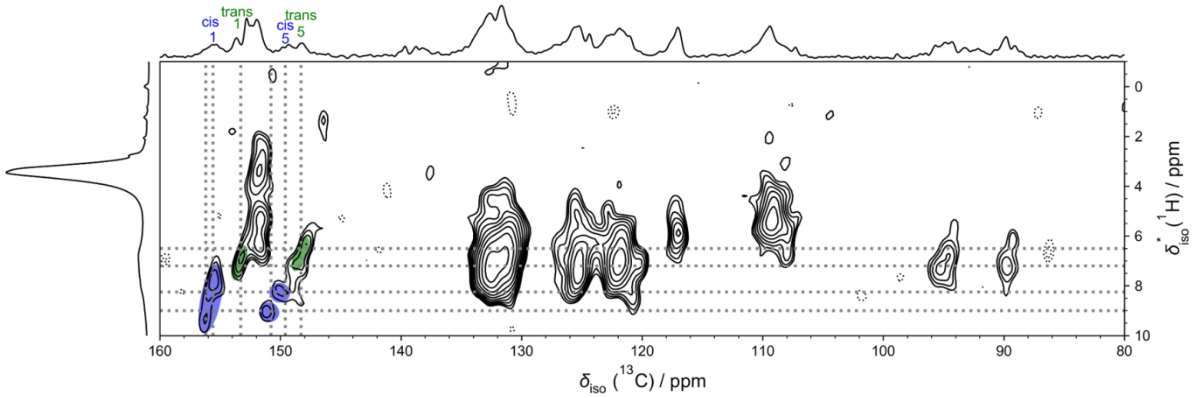

Figure S9: 2D solid-state $^{13}C\{^1H\}$ FSLG-LGCP HETCOR NMR spectrum for **1** recorded at 7.4 T employing 11.0 kHz MAS and a LGCP contact time of 0.5 ms. The projection in the direct dimension shows the $^{13}C\{^1H\}$ CP/MAS NMR spectrum recorded with a CP contact time of 1.0 ms. The projection in the indirect dimension shows the ^1H MAS NMR spectrum at 11.74 T recorded with 27.778 kHz MAS frequency.

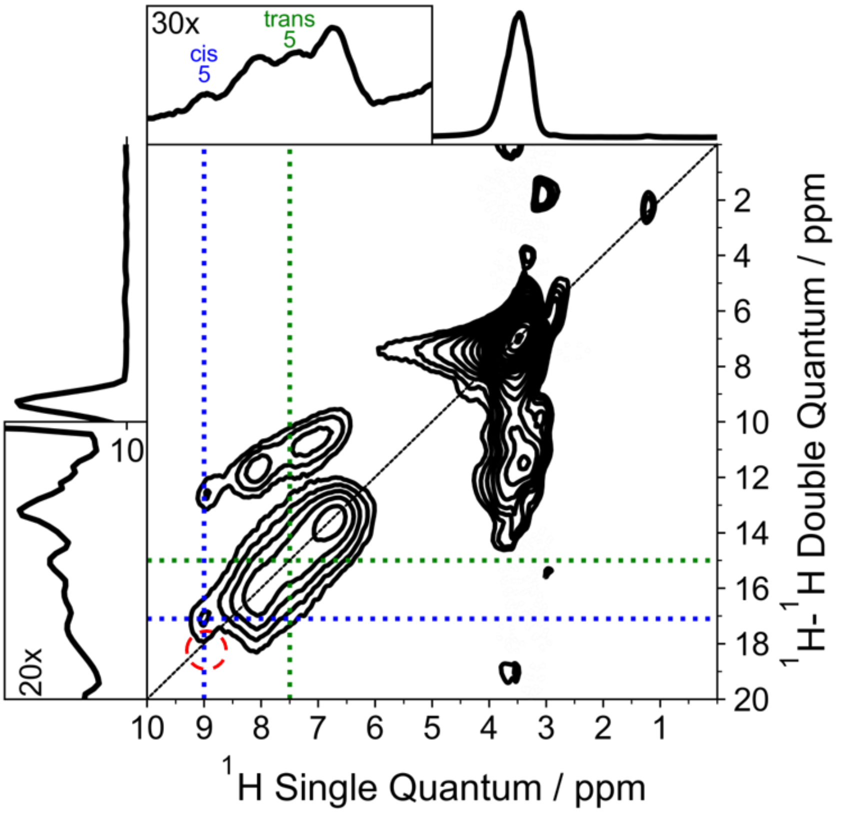

Figure S10: 2D solid-state ^{1}H-^{1}H DQ-SQ NMR correlation spectrum for **1** recorded with 4 rotor periods of Back-to-Back (BaBa) DQ excitation and reconversion at 11.74 T employing 27.778 kHz MAS.

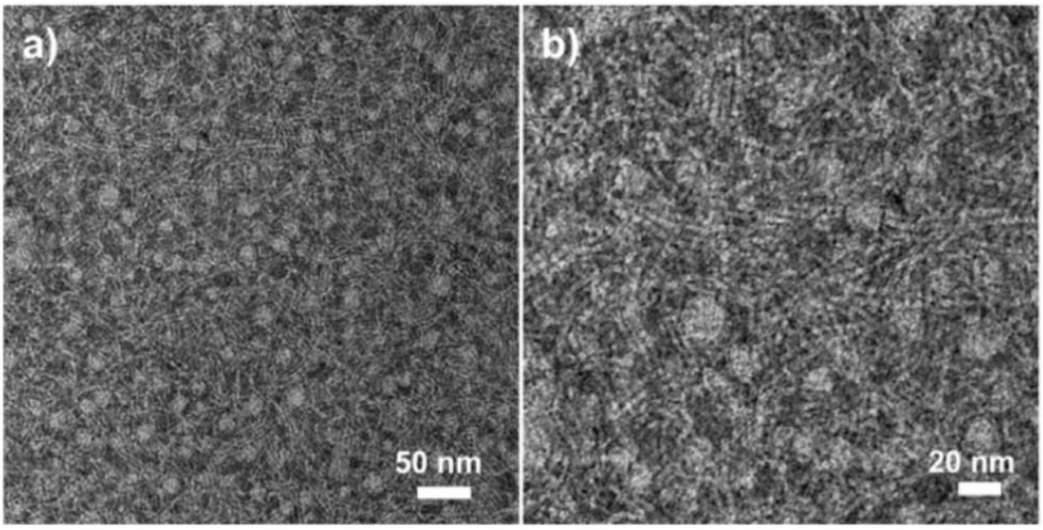

***Figure S11**:* TEM images of solution of **1** in water (5.7 x 10^{-4} M) drop-casted onto a carbon coated copper grid.

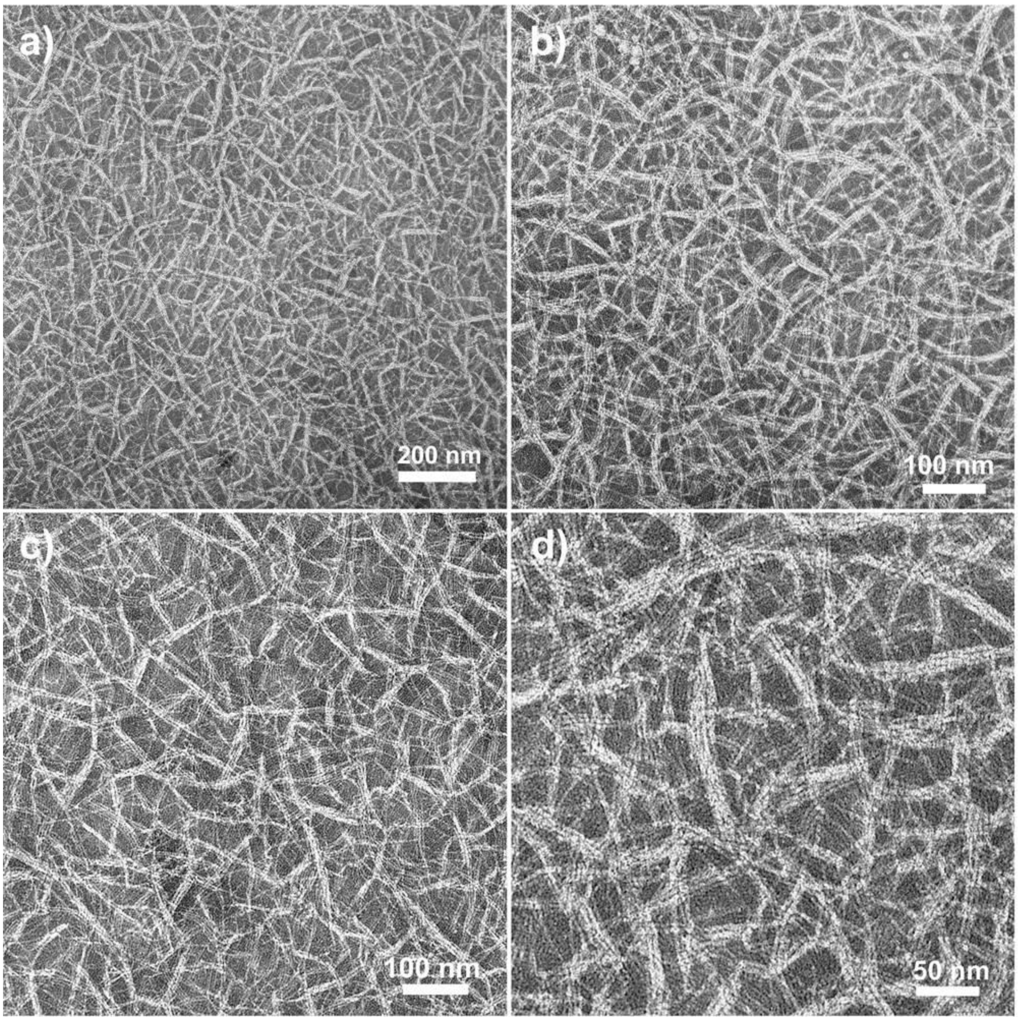

***Figure S12**:* TEM images of **1** in water (8.0 x 10^{-4} M) on a carbon-coated copper grid.

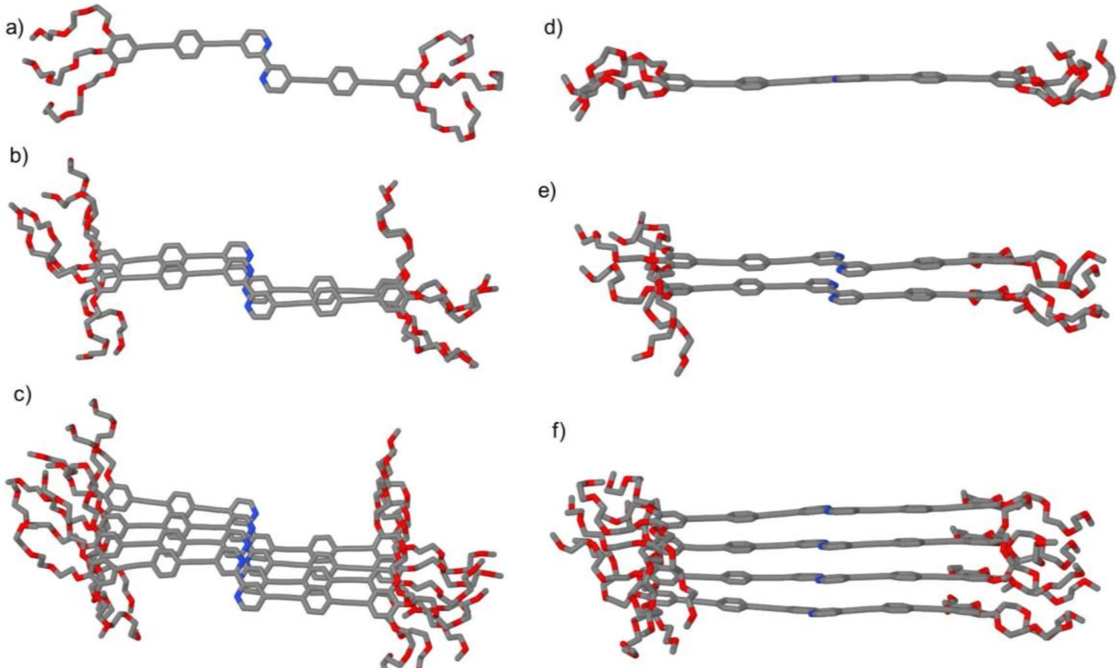

***Figure S13**:* Optimized geometries of the (a, d) monomer, (b, e) dimer and (c, f) tetramer of **1** (a, b, c represent top view and d, e, f represent side view).

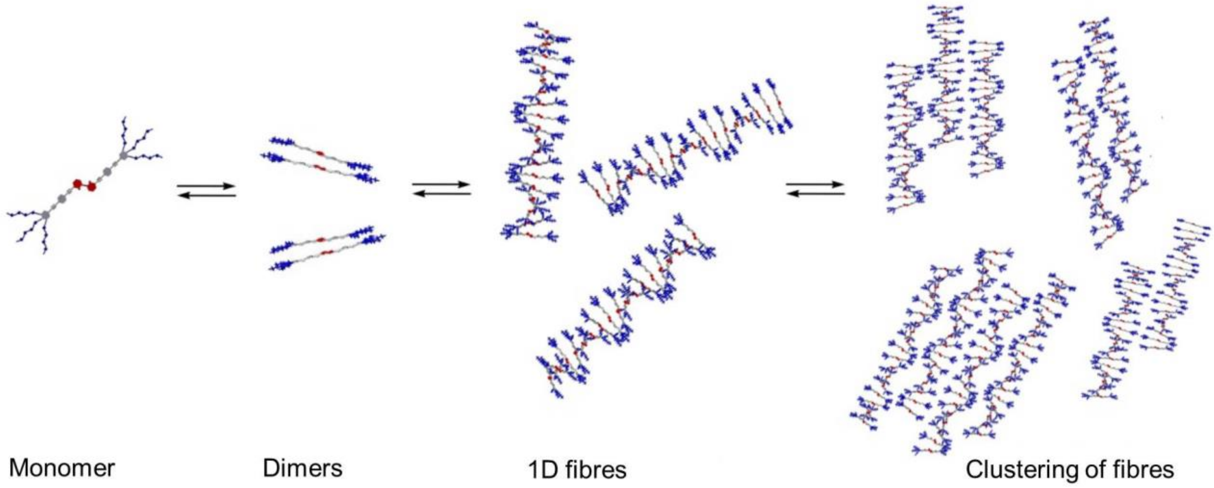

Figure S14: Cartoon representation of the proposed hierarchical aggregation pathway of **1** in aqueous solution.

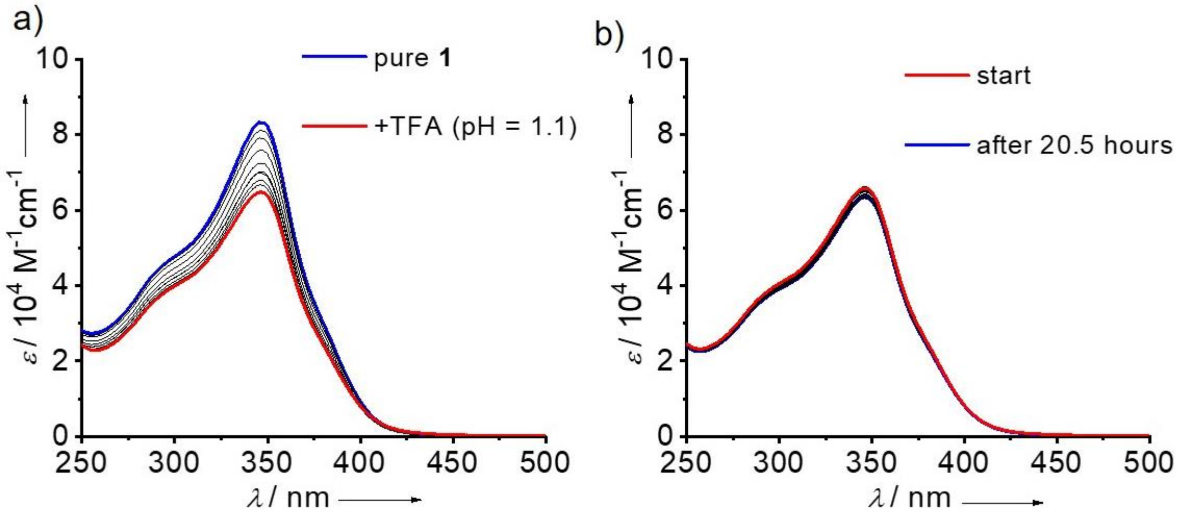

Figure S15: a) Protonation studies of **1** in water/THF = 99:1 (1.5 x 10^{-5} M) upon addition of TFA down to pH = 1.1. b) Protonation studies of **1** in water/THF = 99:1 (1.5 x 10^{-5} M). The spectra show the time-dependent investigation of the solution at pH = 1.1.

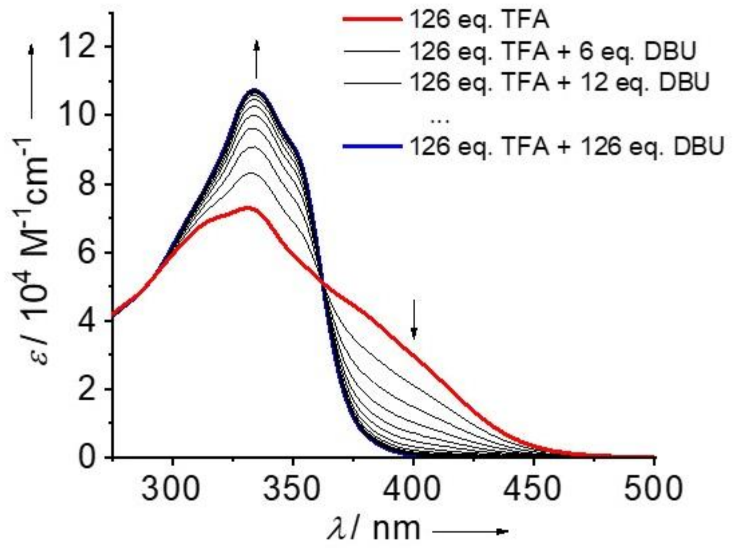

Figure S16: Deprotonation of **1-H⁺** in acetonitrile (1.5 x 10^{-5} M) using DBU as base in acetonitrile at a ratio of 1:24.

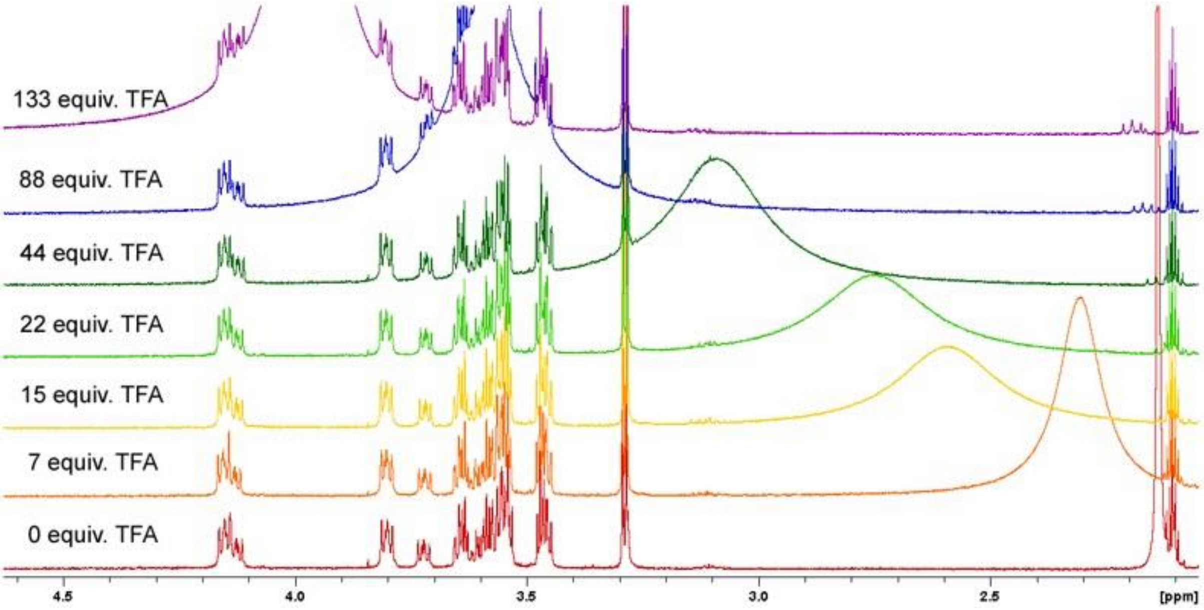

Figure S17: ^{1}H NMR protonation studies of **1** in CD_3CN (to yield **1-H⁺**) upon increasing amount of TFA from 0 to ~133 eq. Partial ^{1}H NMR spectra showing the protons corresponding to the glycol chains that remain unaffected during TFA addition.

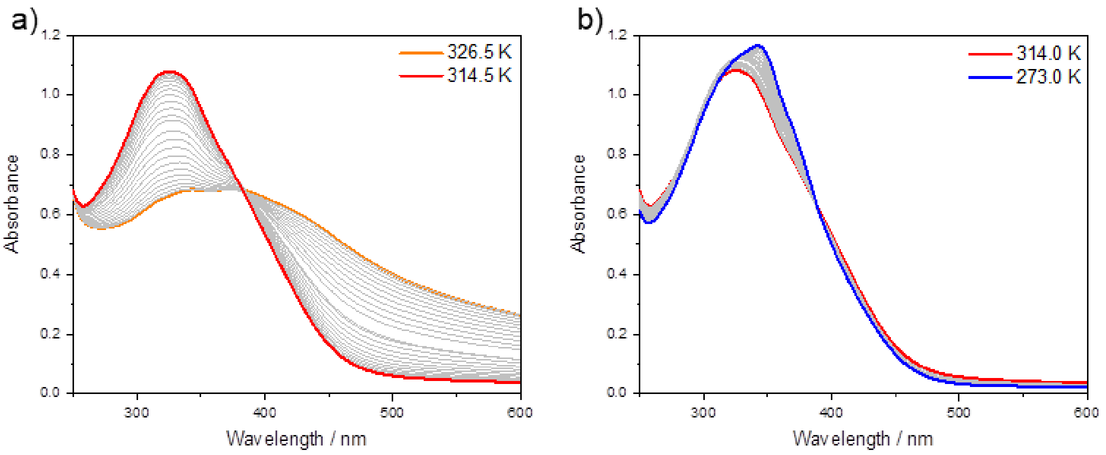

Figure S18: VT UV-Vis experiments of **1-H$^+$** (2×10^{-5} M) in THF/water mixture using 90 % 0.1 M TFA in water and 10 % 1.0 M TFA in THF. a) Dissolution of the precipitate with simultaneous deprotonation (326.5 to 314.5 K) and b) subsequent aggregation of the free ligand **1** (314.0 to 273.0 K).

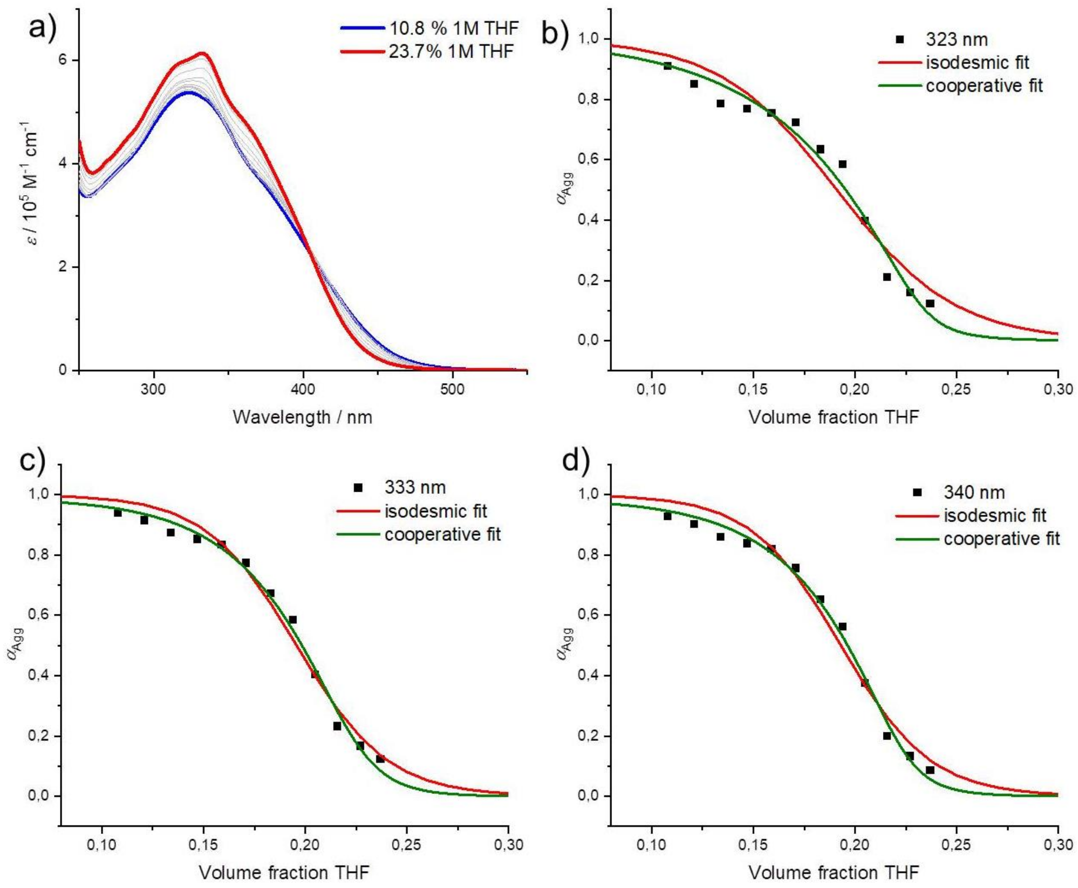

Figure S19: a) UV-vis spectra of **1-H$^+$** in aqueous TFA solution (0.1 M) (c = 2 x 10^{-5} M, aggregted state) upon subsequent addition of **1-H$^+$** in a good solvent (TFA in THF (1.0 M), c = 2 x 10^{-5} M, monomeric state) in small steps. b-d) Denaturation of the aggregate as plot of the volume fraction of good solvent (THF) against the degree of aggregation (α) for b) 323 nm (λ_{max} aggregate), c) 333 nm (λ_{max} monomer) and d) 340 nm. For illustrative purposes the obtained data was fitted to the isodesmic (red line) and cooperative (green line) respectively.

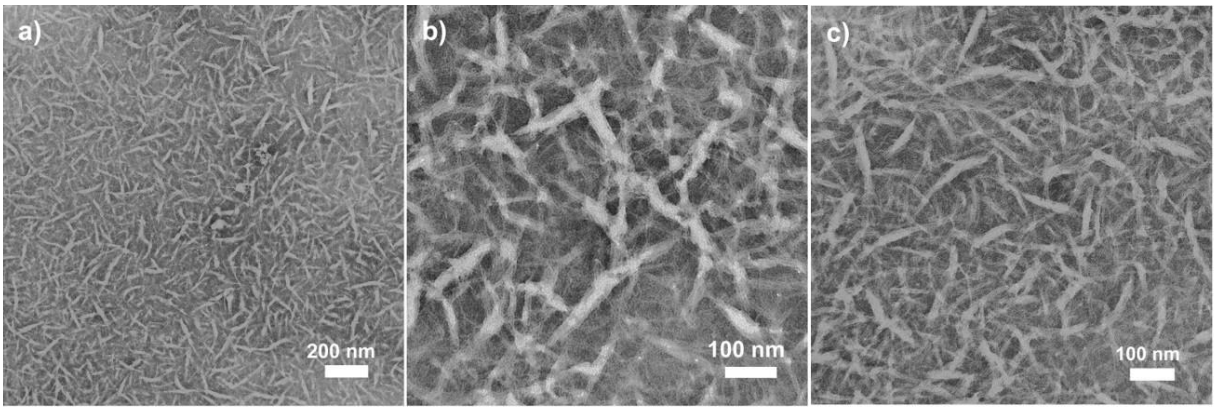

Figure S20: TEM images of aggregated **1-H⁺** in water (7.1 x 10^{-4} M) drop-casted onto a carbon-coated copper grid.

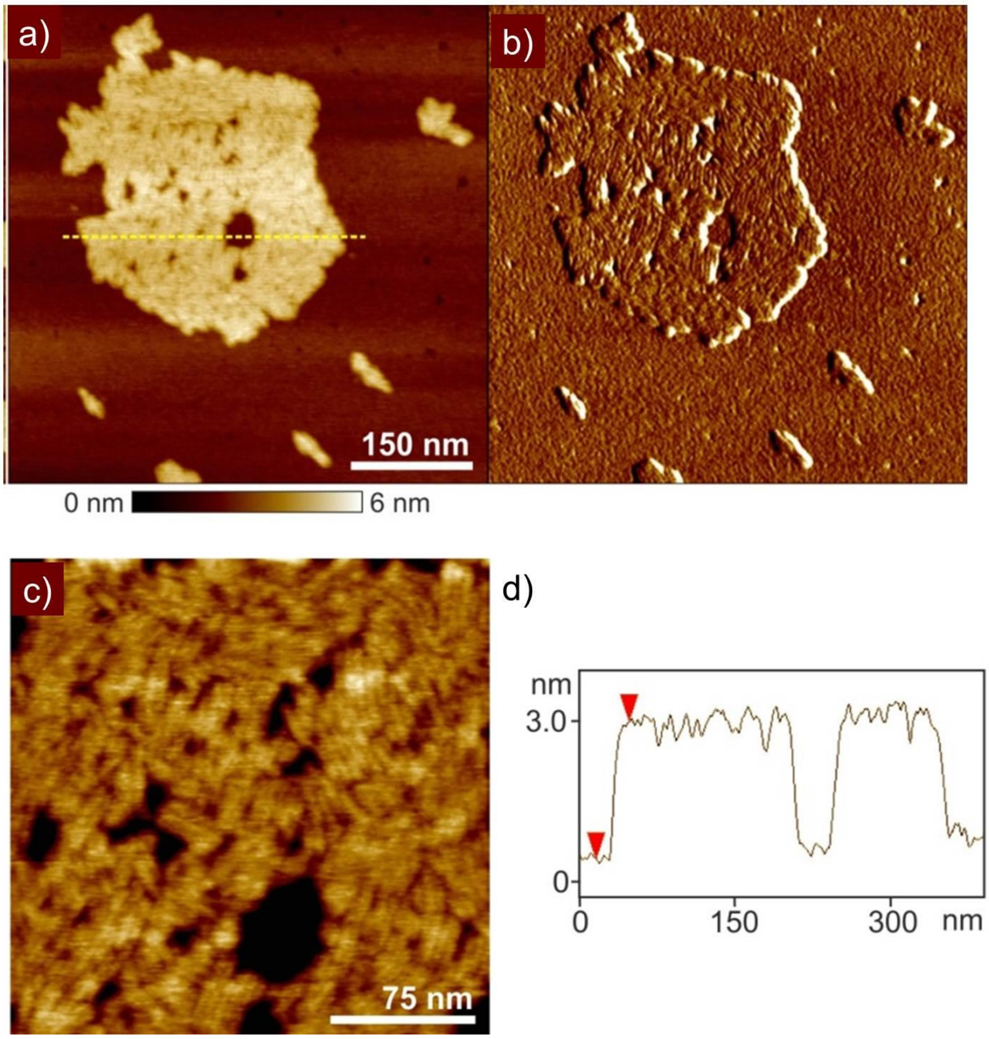

Figure S21: AFM height (a, c) and phase (b) images of **1-H⁺** in water (7.1 x 10^{-4} M). The samples were prepared by spin-coating the solution with 7000 rpm onto mica. Image (d) shows the cross section analysis along the yellow line in image (a).

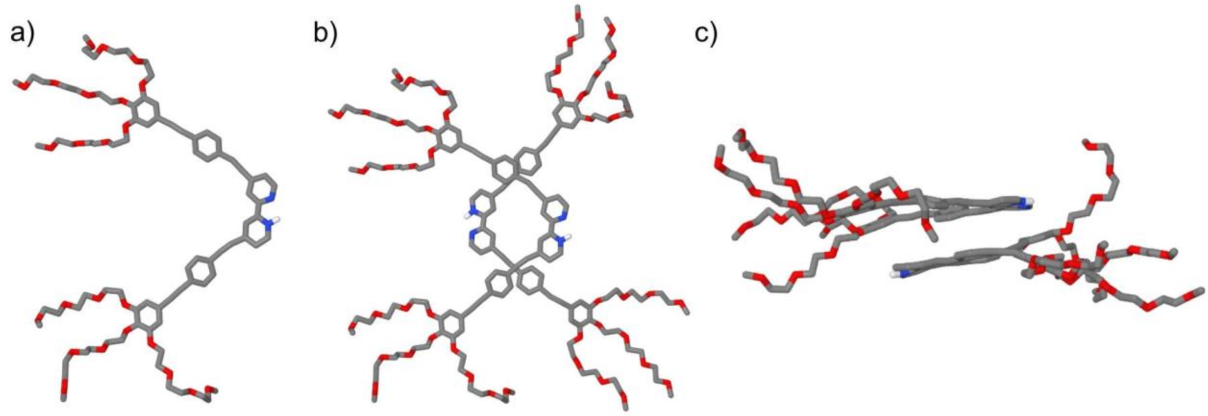

Figure S22: Optimized geometry of the a) monomer and (b, c) dimer of **1-H⁺** (b represents top view, and c represent side view of the dimer).

D. References

[S1] S. Hayashi, K. Hayamizu, *Bull. Chem. Soc. Jpn*. **1991**, *64*, 685–687.

[S2] K. Saalwächter, F. Lange, K. Matyjaszewski, C. F. Huang, R. Graf, *J. Magn. Reson*. **2011**, *212*, 204-215.

[S3] M. E. Halse, L. Emsley, *Phys. Chem. Chem. Phys*. **2012**, *14*, 9121.

[S4] C. Vinod Chandran, P. K. Madhu, N. D. Kurur, T. Bräuniger, *Magn. Reson. Chem*. **2008**, *46*, 943–947.

[S5] D. Massiot, F. Fayon, M. Capron, I. King, S. Le Calvé, B. Alonso, J. O. Durand, B. Bujoli, Z. Gan, G. Hoatson, *Magn. Reson. Chem*. **2002**, *40*, 70–76

[S6] "Turbomole program package for ab initio electronic structure calculations, http://turbomole.com," **2013**.

[S7] J. Tao, J. P. Perdew, V. N. Staroverov, G. E. Scuseria, *Phys. Rev. Lett*. **2003**, *91*, 3–6.

[S8] F. Weigend, R. Ahlrichs, *Phys. Chem. Chem. Phys*. **2005**, *7*, 3297.

[S9] A. D. Becke, *J. Chem. Phys*. **1993**, *98*, 5648–5652.

[S10] A. Ortega, J. García de la Torre, *J. Med. Phys*., **2003**, *119*, 9914-9919.

[S11] P. A. Korevaar, C. Schaefer, T. F. A. de Greef, E. W. Meijer, *J. Am. Chem. Soc*. **2012**, *134*, 13482-13491.

[S12] M. Korzec, S. Kotowicz, K. Laba, M. Lapkowski, J. G. Malecki, K. Smolarek, S. Maćkowskian, E. Schab-Balcerzak, *Eur. J. Org. Chem*., **2018**, 1756-1760.

[S13] A. Florian, M. J. Mayoral, V. Stepanenko, G. Fernández, *Chem. Eur. J.,* **2012,** *18*, 14957-14961.

[S14] P. V. James, K. Yoosaf, J. Kumar, K. G. Thomas, A. Listorti, G. Accorsi and N. Armaroli, *Photochem. Photobiol. Sci*., **2009**, *8*, 1432-1440.

[S15] P. Jonkheijm, P. van der Schoot, A. P. H. J. Schenning and E. W. Meijer, *Science* **2006**, *313*, 80-83.

[S16] H. M. M. Ten Eikelder, A. J. Markwoort, T. F. A. De Greef, P. A. J. Hilbers, *J. Phys. Chem. B.,* **2012**, *116*, 5291-5301.

[S17] A. J. Maarkvort, H. M. M. Ten Eikelder, P. J. J. Hilbers, T. F. A. De Greef, E. W. Meijer, *Nat. Commun*. **2011**, *2*, 509-517.

[S18] P. A. Korevaar, C. Schaefer, T. F. A. de Greef, E. W. Meijer, *J. Am. Chem. Soc*. **2012**, *134*, 13482-13491.
